# Supplementary material for: Lamellar carbon nitride membrane for enhanced ion sieving and water desalination
Source: Nat Commun. 2022 Nov 29;13:7339. doi: 10.1038/s41467-022-35120-9 (PMC9705542; doi:10.1038/s41467-022-35120-9)
Supplement: Supplementary file 1 — Supplementary Information [file 41467_2022_35120_MOESM1_ESM.pdf]

Supplementary Information

Lamellar carbon nitride membrane for enhanced ion sieving and water desalination

Yang Wang[1*], Tingting Lian[1], Nadezda V. Tarakina[1], Jiayin Yuan[2], Markus Antonietti[1*]

[1]Department of Colloid Chemistry, Max Planck Institute of Colloids and Interfaces, 14476 Potsdam, Germany.

[2]Department of Materials and Environmental Chemistry, Stockholm University, 10691 Stockholm, Sweden.

*Corresponding authors. Email: Yang.Wang@mpikg.mpg.de; Markus.Antonietti@mpikg.mpg.de

This PDF file includes:

Supplementary Methods

Supplementary Notes 1-4

Supplementary Figs. 1-43

Supplementary Tables 1-3

Supplementary References (1-32)

Supplementary Methods

Characterizations

1) X-ray diffraction (XRD) patterns of powders and membranes were recorded on Bruker D8 Advance diffractometer equipped with a scintillation counter detector with CuKα (λ = 1.5418 Å) applying 2θ step size of 0.01° and counting time of 1 s per step. Being different from powder samples, a narrower 2θ range (5-20°) was established to afford quicker test to minimize autonomous drying of membrane in air.

2) Fourier transform infrared (FT-IR) spectra were performed on Thermo Scientific Nicolet iD5 spectrometer (550-4000 cm^{-1}) equipped with an attenuated total reflection unit applying a resolution of 0.5 cm^{-1}.

3) X-ray photoelectron spectroscopy (XPS) measurements were carried out on a Thermo ESCALab 250Xi high-performance electron spectrometer using monochromatized Al Kα radiation (hν = 1486.6 eV) as the excitation source. Ar-ion sputtering was applied for the XPS depth profile analysis (50, 100, 150 and 200 nm) of ACN-3 membrane, the power of ion-gun source was 4000 V × 140 μA and etching area was 2.5 mm × 2.5 mm.

4) Atomic force microscopy (AFM) analysis was performed on Veeco Dimension 3100 atomic force microscope. Specifically, the CN supernatant was diluted, dropped onto cleaned mica surface and dried overnight at room temperature prior to test. In terms of thickness and roughness measurements, CN membrane was fabricated and subjected to a membrane transfer process[1,2] to avoid the potential interference from PDA-PES filter. Specifically, CN suspension was filtrated onto mixed cellulose ester (MCE) filter (pore size: 0.2 μm, ADVANTEC, Toyo Roshi Kaisha, Ltd., Japan), followed by drying at 60 °C under vacuum for > 15 h. The membranes were then immersed in acetone bath to dissolve MCE, the freestanding membrane fragment was further washed five times using fresh acetone bath to completely remove MCE. Finally, the cleaned fragment was transferred to silicon wafer (prewashed with water, methanol and purified with high-purity nitrogen gas). The dried membrane fragment was firmly adhered onto silicon wafer without slipping or warping upon solvent evaporation. For ACN membrane deposited on MCE filter, it turned to be fragmentized after liquid nitrogen treatment, due to its poorer affinity to MCE. The fragment however can be advantageous to direct morphology observation.

5) Scanning electron microscopy (SEM) analysis was carried out on a LEO 1550-Gemini microscope equipped with Oxford Instruments EDX detector. Both CN and ACN suspensions were diluted and then dropped onto carbon substrate, followed by drying at 60 °C under vacuum for 1 h before analysis. Note that the ACN suspensions were prepared as follows: The dried ACN powder was re-dispersed in water, followed by 30 min sonication to afford homogenous suspension. This is emphasized because the freshly prepared ACN suspension after aging boils down to a mixture without removing excess Al species, which can not be used for SEM study. For the cross-sectional observation of membrane morphology, the membrane transfer process and fragmentization process for AFM study was again applied for CN and ACN membranes, respectively. This is due to the fact that the brittle fracture of PDA-PES filter is more difficult in liquid nitrogen.

6) Transmission electron microscopy (TEM) study was performed using double Cs corrected JEOL JEM-ARM200F operated at 80kV and equipped with a cold-field emission gun. A suspension of the sample in water was sonicated for 10 minutes and then drop-casted to the Cu grid with a lacey carbon support and dried for 15 minutes.

7) Zeta-potential measurements were completed using Malven Instruments (Zetasizer Nano ZS ZEN 3500, MAL 500329). Particularly, the purified ACN powder was redispersed in water by sonication for 30 min to afford suspension. The zeta-potential variation as a function of pH value (adjusted by 1M HCl or NaOH solutions) was also measured for the purified ACN-3 suspension.

8) Contact angle tests were carried out using sessile drop analysis (OCA measurement, Dataphysics ES, Germany) and processed via the official Krüss software. The membranes were adhered onto double-sided adhesive tape and further cohered on glass substrate to afford flat surface. A known volume of 10 μL water was gently dropped onto membrane surface, at least three different spots on the membrane were measured.

9) Liquid-state ^{27}Al-nuclear magnetic resonance (NMR) spectra were collected on JEOL JNM-ECZ600R/S1 spectrometer with the following parameters: single-pulse method, resonance frequency of 156.38 MHz, 90¨ pulse width of 12 μs, π/6 pulse, sampling interval of 2 s for 1000 scans.

10) ^{1}H cross-polarization/magic angle spinning solid-state nuclear magnetic resonance (CP/MAS ssNMR) experiments were performed on a Bruker AVANCE III 400 WB spectrometer operating at 100.62 MHz for 1H using a double resonance 4 mm MAS NMR probe and a sample spinning rate of 8 kHz.

11) Nitrogen sorption isotherms were recorded at 77 K using a Quantachrome Quadrasorb SI apparatus. Prior to the measurements, the samples were activated at 120 ºC for 20 h under dynamic vacuum (0.5 Torr, 3P Instruments Masterprep degassing device). The Brunauer-Emmett-Teller (BET) method was applied to calculate the specific surface area from adsorption branch data ($0.05 < P/P_0 < 0.35$). The pore volume was calculated from the amount of gas adsorbed at $P/P_0 = 0.995$.

Supplementary Note 1

Strategy for nanochannel activation of pristine CN coupled with conformal packing

Pristine CN represents a thermally polymerized product of carbon- and nitrogen-rich precursors (melamine, cyanamide, dicyanamide, urea, *etc.*)[3]. The combined merits of easy preparation, tunable electronic structure, visible-light responsiveness and high stability impart CN with exciting opportunity for solar energy conversion when serving as a metal-free photocatalyst[4]. The electron-rich nature arising from *sp*^{2}-hybridized nitrogen and amino groups entitles CN to abundant Lewis basic sites[5]. In this regard, alternative acidic candidates with weak oxidizing or corrosive properties but strong deprotonation ability are expected to afford acid-base reactions whilst maintain the conjugated framework of CN. Keggin-type polyaluminum clusters with high cationic charge and abundant hydroxyl/aquo ligands on the surface are extensively used for catalysis and clarifying agents for water purification[6], such as $[Al_{13}O_4(OH)_{24}(H_2O)_{12}]^{7+}$ (Al_{13}) and $[Al_{30}O_8(OH)_{56}(H_2O)_{26}]^{18+}$ (Al_{30}) displayed in Supplementary Fig. 1. Al-Ferron kinetic study (Supplementary Fig. 2) and liquid-state ^{27}Al NMR spectra (Supplementary Fig. 3) point to the

successful synthesis of the two clusters. Al_{30} bearing a wide variety of surface functional groups[6], especially the 26 acidic bound waters with asymmetric distribution (Supplementary Fig. 1b), is more acidic than Al_{13} with 12 symmetrically identical water molecules at terminal positions (Supplementary Fig. 1a). The most acidic group ηH_2O-4 (Supplementary Fig. 1b) strongly couples with the adjacent ηH_2O-3 as site-specific contributions for deprotonation, the relative Bronsted acidity of these groups falls in the sequence of (ηH_2O-3 + ηH_2O-4) > ηH_2O-5 > ηH_2O-1 > ηH_2O-2[7], in accordance with our calculation results (Supplementary Fig. 4). Note that the bridged OH groups are far less reactive and only come with minimal contributions for deprotonation, they are not discussed herein. As such, the interaction between Al_{30} and CN shares much higher adsorption energy relative to that of Al_{13} and CN (-7.3 eV *vs.* -1.4 eV, Supplementary Fig. 5), and the final Al_{30}-CN composite is characterized by the most acidic ηH_2O groups of Al_{30} lying flat on the CN plane.

The negatively charged CN nanosheet, when protonated by Al_{13} in varied proportions, only gives slight changes of zeta potentials shifting towards positive direction (Supplementary Fig. 6a). The XRD patterns of Al_{13}-CN composites also closely resemble that of pristine CN nanosheet (Supplementary Fig. 6b). Collectively, Al_{13} as an efficient high-charge pillar with lower steric hindrance than Al_{30}, turns out to be unsuccessful for CN activation. Comparatively, highly acidic Al_{30} with larger cluster size substantially neutralizes the negative charge of CN nanosheet even at very low proportion (Al_{30}:CN = 0.025:1, wt%), and further plateaus the zeta potential values (~ +33 mV) of Al_{30}-CN (ACN) composite at higher concentrations (Supplementary Fig. 7). Activated CN by Al_{30} is then exemplified by the XRD patterns (Fig. 2a in main text) with broadened interlayer spacing. Bulk CN features multilayered microstructure and turns into few-layer nanosheets upon sonication-assisted exfoliation and purification (Supplementary Fig. 8), statistical analyses of the nanosheet size and height suggest that the thickness of these CN nanosheets was around 1.1-1.3 nm, *i.e.*, 3-4 layers (single-layer thickness: ~ 0.32 nm) with lateral size of ~ 300 nm (Supplementary Figs. 8c- 8d). Collecting the powder by centrifuging the CN nanosheet supernatant after aging, however, results in aggregated particles with varied sizes (Supplementary Fig. 9a). The random restacking behavior can be detrimental in terms of assembling 2D CN membrane where layer-by-layer packing pattern is of necessity. Impressively, the ACN composite powder gradually evolves from random particles to well-ordered laminates with increasing Al_{30} amount (Supplementary Figs. 9b-9h), and the layers are partially swallowed when excess Al_{30} was introduced (Supplementary Fig. 9i). The significant difference between CN and ACN signifies that Al_{30} clusters act as pillars to afford conformal packing of CN nanosheets, and the homogeneity of ACN-3 (CN: Al_{30} = 1: 2, wt%) composite powder is confirmed by EDX-mapping results (Supplementary Fig. 10, chlorine exists as counterion in Al_{30}). The nitrogen sorption isotherms (Supplementary Fig. 11) verified that both surface area and pore volume increased with increasing Al_{30} amount, again pointing to the effective pillaring effect from the polycations. Further increasing Al_{30} resulted in the decrease of surface area and pore volume in ACN-4, this can be ascribed to the blocked pores by Al_{30}, which tended to make the lamellar structure aggregated and less identifiable (Supplementary Fig. 9i). It should be emphasized that the nitrogen sorption data cannot be used to determine the interlayer channel for molecules or ions to transport in 2D membranes, instead, it only indicates the existence of available spaces for foreign species to access. Taking together, Al_{30} cluster as pillaring agent captures the dual advantages for nanochannel activation and ordered layer stacking of CN, both of which are recognized challenges for CN membranes to be applied in precise ionic and molecular separation enabled by tunable interlayer spacing based on a 2D structure.

Supplementary Note 2

Noncovalent interaction between CN and Al_{30}

Pristine CN in both bulk and nanosheet forms show characteristic peaks at 802 cm^{-1}, 1200-1700 cm^{-1} and 3000-3300 cm^{-1} (Supplementary Fig. 13) assigned to the out-of-plane bending vibration of tri-s-triazine ring, stretching vibration of aromatic C-N and C=N heterocycles, and stretching vibration of N-H and adsorbed O-H groups, respectively[8]. Specifically, a set of peaks at 1198, 1224, 1312, 1393, and 1452 cm^{-1} are indicative of C-N stretching vibrations, while the two peaks at 1538 cm^{-1} and 1621 cm^{-1} are C=N stretching vibrations[9]. Upon intercalation by Al_{30}, theses vibrations are retained and shift towards high wavenumbers, but show progressively decreased intensity with increasing Al_{30} amount. Exceptional case is identified for C-N stretching, which shifts from 1224 cm^{-1} to 1250 cm^{-1} but with intensified signal. The red shifts of these peaks and better resolved C-N vibration peak are attributed to the protonation effect Al_{30}, which donates protons to the electron-rich nitrogen in CN and forms N---H-O hydrogen bonding[10]. The existence of Al_{30} in the composite is evidenced by several bands: the declinate peaks at 700-780 cm^{-1} and 910-1050 cm^{-1} can be associated with the stretching vibration of Al-O in [AlO_4] tetrahedra and [AlO_6] octahedra, respectively[27,32]. Besides, the stretching vibration of N-H and marginal O-H in pristine CN is now dominated by that of O-H at 3200-3500 cm^{-1} in Al_{30}-CN samples, which is interpreted as a mixture of bound water molecules and bridged OH groups from Al_{30}[6,11]. The broad peak also shifts towards higher wavenumbers with increasing Al_{30} amount, again suggesting the interaction between Al_{30} and CN is dominated by hydrogen bonding[12]. Note that the N-H group of pristine CN is retained (will be revealed by XPS spectra afterwards) in the composite but largely overlapped by the broad peak of O-H. The band evolution indicates the interplay between Al_{30} and CN is governed by non-covalent interactions.

The chemical bonding environment is also revealed by XPS results (Supplementary Fig. 14). The two peaks at 288.3 eV and 284.8 eV in the C 1*s* spectra of CN bulk and nanosheet stem from sp^2 bonded C (N-C=N) and standard reference carbon, respectively[2]. In ACN samples, the peak shifts from 288.3 eV to 288 eV and a new peak assigned to C-N emerges at 286.3 eV[13]. The N 1*s* spectra of CN samples can be deconvoluted into four peaks at approximately 398.8, 400.1, 401.2 and 404.4 eV, which are indicative of sp^2 hybridized N (C-N=C), ternary nitrogen (C-$(N)_3$), amino groups (C-NH_x, x = 1 or 2) and charging effects in the aromatic heterocycles, respectively[14]. Again, these peaks shift towards lower binding energies (398.5, 399.9, 401 and 404.1 eV) in ACN samples. The combined information in C 1*s* and N 1*s* spectra points to the strong interaction between Al_{30} and CN. Relative either to C-N=C or C-$(N)_3$, the proportion of C-NH_x groups experiences progressive decrease upon increasing Al_{30} amount (Supplementary Table 1). This is in sharp contrast to the evidently increased C-NH_x proportion in strong corrosive acid (*e.g.* concentrated H_2SO_4) and base (*e.g.* KOH) treated CNs, where they act more like scissors to fragmentize the conjugated framework by hydrolysis[15,16]. Accordingly, we assume the new peak in C 1*s* spectra (286.3 eV) is assigned to the nitrogen of sp^2 C-N=C with its long-pair electron to accept proton and forms C-NH=C, rather than the edge C-NH_x. The only peak at 532.6 eV in the O 1*s* spectra of CN samples is likely caused by water adsorption, which however evolves into two different peaks at 532 eV and 530.7 eV assigned to -OH and Al-O of Al_{30}, respectively[17]. In the Al 2*p* spectra of ACN composite, the two peaks at 74.3 eV and 73.7 eV are associated with [AlO_6] octahedra and [AlO_4] tetrahedra, respectively[18]. The ratio of [AlO_6]/[AlO_4] is in close proximity to 14:1, in line with its structural configuration displayed in Supplementary Fig. 1. Besides, the existence of

chlorine is confirmed by the Cl 2p spectra of ACN samples, which is deconvoluted into Cl 2$p_{1/2}$ and Cl 2$p_{3/2}$ at 199.3 eV and 197.8 eV, respectively[19].

Taking together, no covalent interaction between Al_{30} and CN is identified, they prefer to proceed in a non-covalent manner. Specifically, Al_{30} first serves as charge-compensation agent to neutralize the negative charge of CN by electrostatic attraction and concurrently pillars in-between adjacent CN layers, which further enables excellent dispersibility of the ACN composite (zeta potential variations in Supplementary Fig. 7) at higher loading amount, by virtue of electrostatic repulsion of the positively charged ACN. Our FTIR and XPS results reveal that the sp^2-hybridized nitrogen in CN allows such an interplay between Al_{30} and CN, which is dominated by protonation-enabled interlayer hydrogen bonding (Fig. 1 in main text).

Supplementary Note 3

<u>Membrane stability</u>

As an initial try, we dried the ACN-3 membrane at 20 °C in vacuum, and probed its stability in water (Supplementary Fig. 19). Unfortunately, the membrane soon detached from the PDA-PES filter and got disintegrated, similar to that of graphene oxide membrane deposited on polymeric substrate[20]. Consequently, the colorless water turned turbid. There were probably some residual ACN components left on the filter, but the XRD pattern only shows the peak assigned to PDA-PES filter. Upon elevating the temperature to 60 °C and 100 °C, ACN membrane is quite stable when immersed in water for 24 h. Neither disintegration nor serious swelling was observed, as suggested by the digital photos and subtle fluctuation of the membrane in wet state.

In a next step, we evaluated the water contact angles of as-prepared membranes dried at different temperatures (Supplementary Fig. 20). Pristine CN membrane maintains its hydrophilicity over the wide temperature window (20 °C, 60 °C and 100 °C). With increasing Al_{30} amount, the ACN membranes dried at 20 °C are characterized by decreased hydrophilicity relative to that of CN, which are more evident at 60 °C. Specifically, all the ACN membranes with higher Al_{30} amount turn to be hydrophobic (> 90° for ACN-2, ACN-3 and ACN-4). Further increase of temperature to 100 °C even entitles the ACN-1 membrane with lowest Al_{30} loading amount to hydrophobic surface, and the other ACN membranes maintained the hydrophobicity at this temperature that is acquired at 60 °C already. Taking the ACN-3 membrane as a typical example, we found the water contact angle was 68° after drying at 20 °C, a much less hydrophilic surface than that of CN membrane. The disintegration of ACN-3 suggests that the removal efficiency of adsorbed water and dehydroxylation of some loosely bonded OH groups is quite low at this temperature (Fig. 2e in main text), which retains in the sub-nanochannel and tends to decompose the membrane on hydration. Elevating the temperature to 60 °C and 100 °C helps to eliminate the dilemma *via* removing the weakly-bonded water or OH groups in ACN layers, accompanied by switching membrane surface from hydrophilic to hydrophobic. Benefited from the well interlinked nanostructure, these membranes are stable against disintegration or serious swelling in water environment, although bound water molecules and bridged OH groups are retained in ACN. As the ACN membrane dried at 100 °C contributed marginally to advance the ion rejection performance, we will not discuss more hereafter.

The possible leaching of Al species from ACN-3 membrane immersed in water was excluded, as we observed no obvious conductivity change of the solution under stirring for 24 h (Supplementary Fig. 21). Prior to ion permeation test, we also evaluated the stability of ACN-3 membranes in salt solutions. Upon drying at 60 ℃ under vacuum, the membranes were immersed in a wide variety of aqueous salt solutions for 24 h. The diffraction peaks show minute shift towards lower angles (Supplementary Fig. 22) and shares similar results with the cases in water-only environment (Fig. 3e in main text), again pointing to the high stability of our membrane and making the permeation test possible and reliable.

Furthermore, the chemical bonding environments of both CN and ACN-3 membranes are in close proximity to their powder counterparts (Supplementary Fig. 14 and Supplementary Fig. 23), detailed analyses are provided in Supplementary Note 2. We used Ar^+ sputtering to acquire the XPS depth profiles of ACN-3 membrane, and found that the elements (C, N, O, Al and Cl) are characterized by homogenous distribution with the etching depth ranging from 0 nm to 200 nm along membrane thickness direction (Supplementary Fig. 24). Prior to ion permeation test, we found that the ACN-3 membrane shows lower salt adsorption capacity relative to CN membrane, probably indicating its higher ion rejection (Supplementary Fig. 25).

Supplementary Note 4

<u>pH-dependent permeation behavior in ACN membrane</u>

Note that the XRD patterns of ACN-3 membrane immersed in pH =12 and pH =2 solutions kept identical with that in Milli-Q water (pH = 5.7), suggesting that ACN retains its structural integrity in both alkaline and acidic environments (Supplementary Fig. 41). Besides, the membrane barely alters its hydrophobicity in these conditions, as the water contact angles highly resemble each other (Supplementary Fig. 42). That is, the free spacing which expands to the same extent under different pH values, or the similar membrane surface in theses environments, is unlikely to be responsible for the tunable water flux or salt rejection performance.

Compared with other representative 2D membranes (*e.g.* graphene oxide, MXene, MoS_2), our ACN-3 membrane holds both high salt rejection and water permeability (Supplementary Fig. 43 and Supplementary Table 3), which turns to be self-adaptive in water environment with a large pH (2 to 12) window and maintains structural integrity.

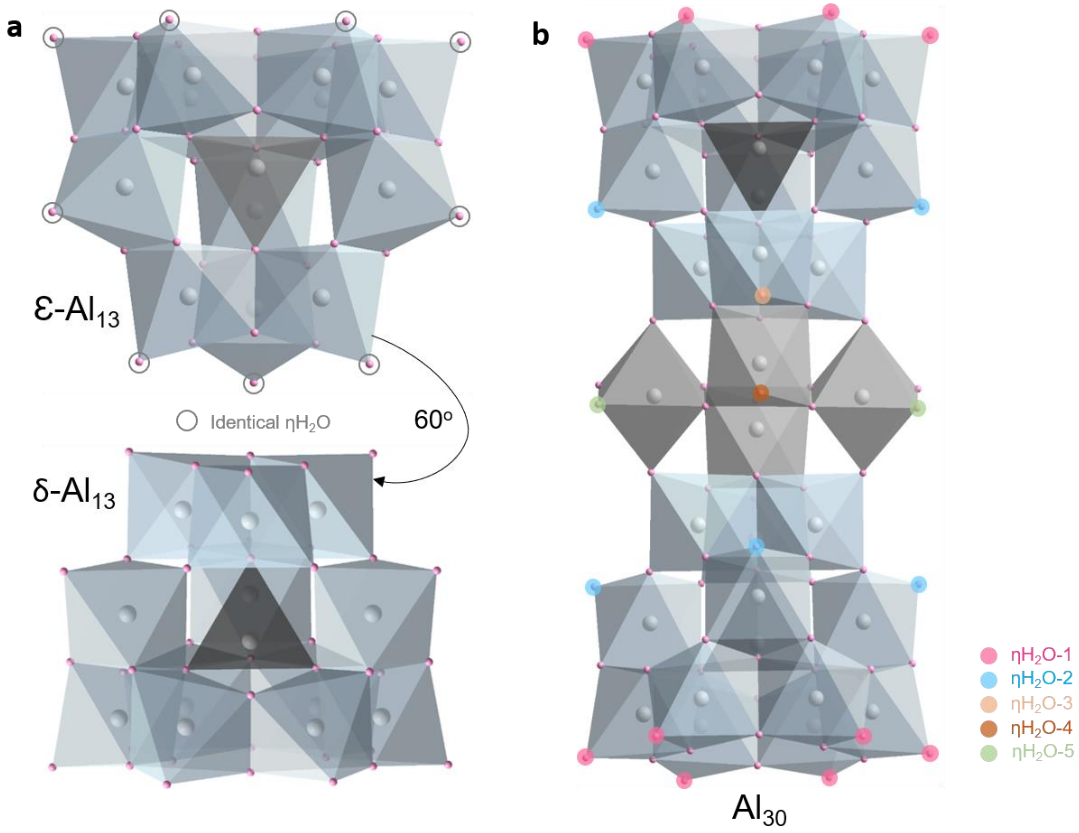

Supplementary Fig. 1 | Structure of (a) Al_{13} and (b) Al_{30}. Al and O atoms are denoted by white and pink balls, H atoms are omitted for clarity. ε-Al_{13} with four [Al_3O_{13}] trimers linked by the central [AlO_4] tetrahedra is thermodynamically favored isomer in Baker-Figgis-Keggin family (α, β, ε, γ, δ). δ-Al_{13} represents another isomer of Al_{13} clusters by rotating one of the [Al_3O_{13}] trimers in ε-Al_{13} for 60°; in this way, it is corner-shared with other isomers rather than edge-shared. Al_{30} comprises two δ-Al_{13} units linked by four [AlO_6] octahedra in the central region. ηH_2O denotes non-bridging bound water molecules, which is identical for ε-Al_{13} (**a**) but grouped into five topological sets (ηH_2O-1, 2, 3, 4, 5) in (**b**) to distinguish their acidities. The other sites are assigned to bridged -OH groups (μ-OH, denoted by pink balls on polyhedrons).

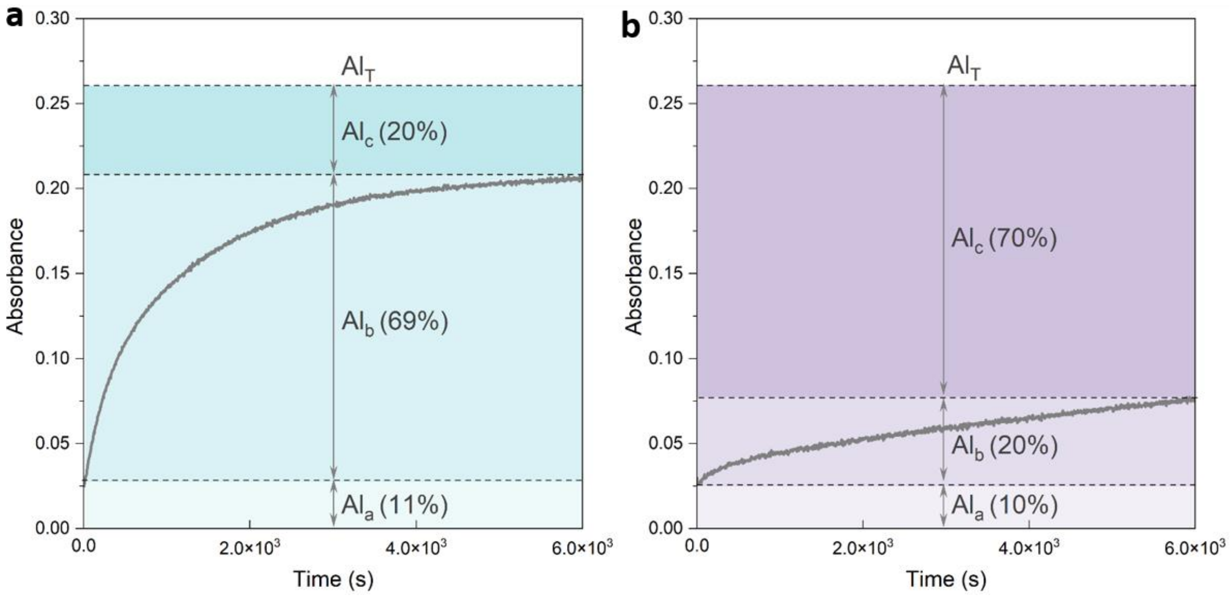

Supplementary Fig. 2 | Al-Ferron kinetics of (a) Al_{13} and (b) Al_{30}. Al_a: mononuclear species that react immediately with Ferron upon mixing, Al_b: low-to-medium polymeric species that experiences slightly more sluggish reaction with Ferron, Al_c: highly polymeric or colloidal species that are non-reactive ($Al_T = Al_a + Al_b + Al_c$). The relative amounts of Al_b in (**a**) and Al_c in (**b**) indicate the high contents of Al_{13} and Al_{30}, respectively.

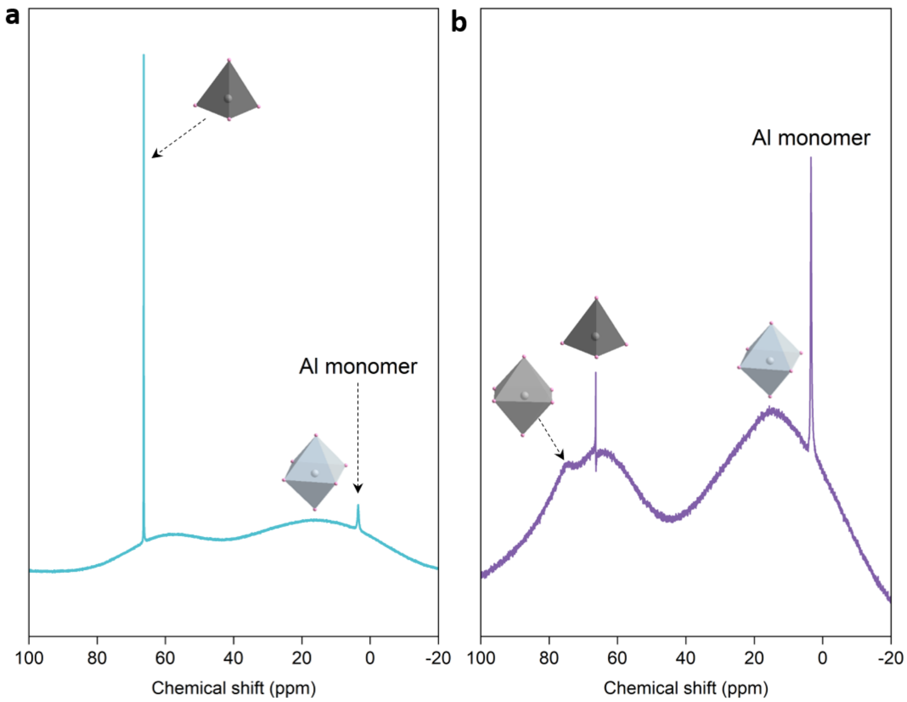

Supplementary Fig. 3 | Liquid ^{27}Al-NMR spectra of (a) Al_{13} and (b) Al_{30}. In both cases, the resonance peaks at 3.5 and 66.4 ppm are assigned to Al monomer and central tetrahedral Al ($[AlO_4]$), respectively[21]. The broader peak window between 10-20 ppm denotes the octahedral Al ($[AlO_6]$) of external Keggin shells in Al_{13} and Al_{30}, caused by the asymmetric coordination environment of $[AlO_6]$ that experiences extremely fast spin relaxation, shortening relaxation time and therefore broadening line width[22]. The new signal around 74.7 ppm in (**b**) is indexed to the central $[AlO_6]$ in Al_{30} and points to the successful synthesis of Al_{30} solution[21]. The two δ-Al_{13} capping units spatially isolated by four $[AlO_6]$ in the central region lowers the structure symmetry of Al_{30} and causes peak shift from 66.4 ppm to 74.7 ppm. The configurations of involved polyhedral in Al_{13} and Al_{30} can be referred to in Supplementary Fig. 1.

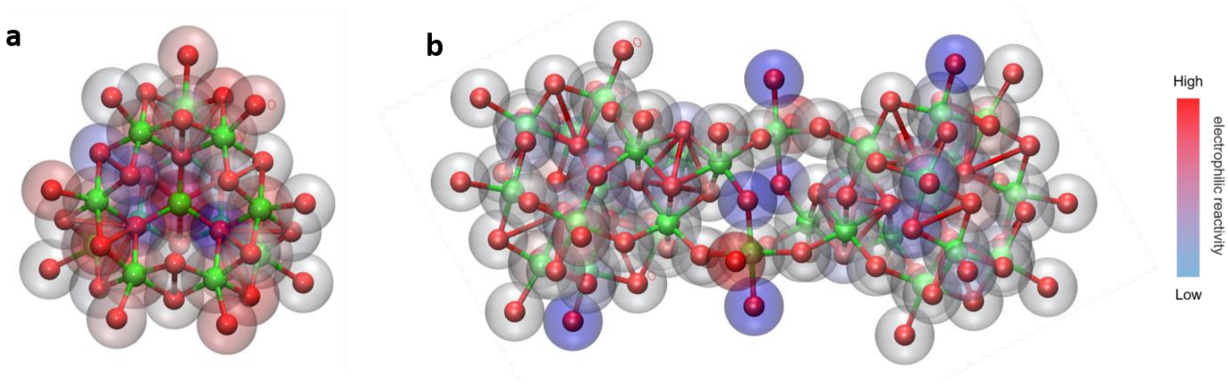

Supplementary Fig. 4 | Fukui index (*f*-) analysis of (a) Al_{13} and (b) Al_{30}. Hydrogen atoms are omitted for clarity, the green and red balls denote Al and O atoms, respectively. In a typical electrophilic process enabled by the protons in the two acidic clusters, the lower reactivity suggests that proton is more likely in a dissociation state and prone to interacting with the long-pair electrons of sp^2-hybridized nitrogen from CN. These low-electrophilic-reactivity sites prefer to centralize in the equatorial region of Al_{30}, pointing to the asymmetric distribution of acidity in Al_{30}.

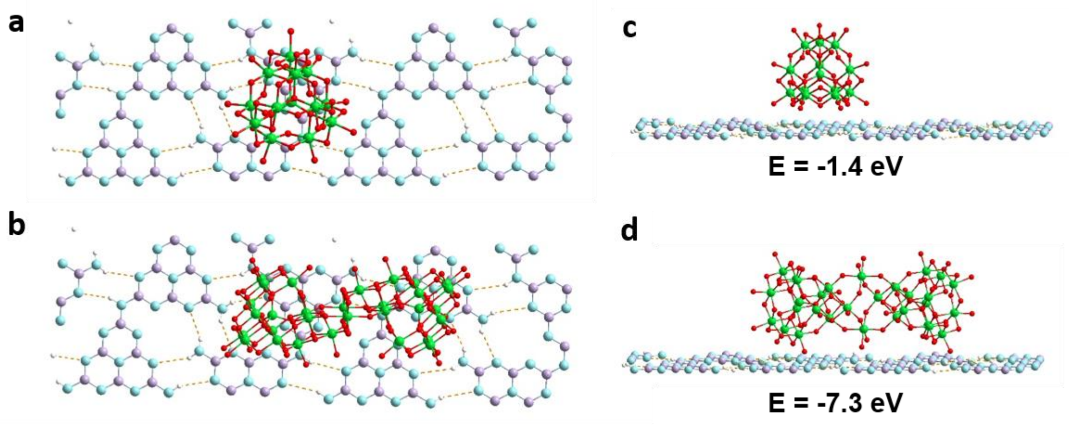

Supplementary Fig. 5 | Theoretical calculations of adsorption models. a, b, Optimized configurations of **a,** Al_{13}-CN and **b,** Al_{30}-CN. **c, d,** Corresponding adsorption energies of **c,** Al_{13}-CN and **d,** Al_{30}-CN. The C, N, Al, O and H atoms are denoted by light purple, light turquoise, green, red and white balls, respectively. The yellow dot lines in CN denote hydrogen bonding.

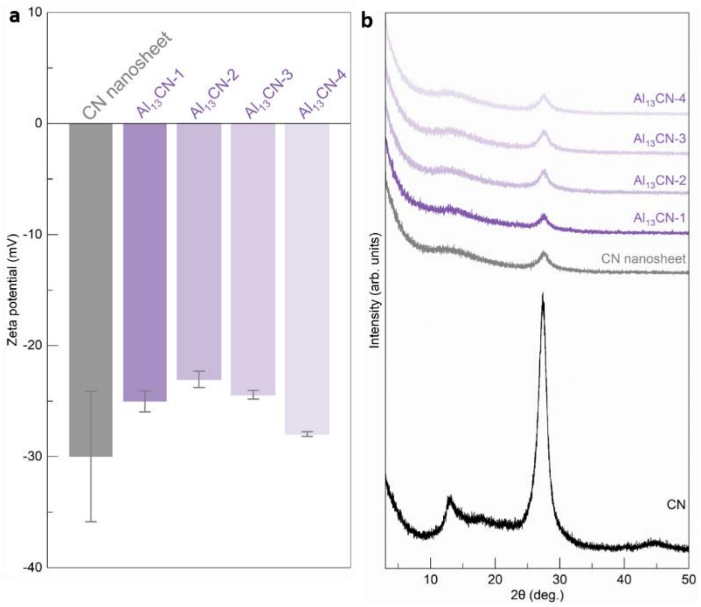

Supplementary Fig. 6 | Zeta potentials (a) and XRD patterns (b) of CN and Al_{13}-CN samples. ($[Al_{13}]/[CN]$ = 0.25:1, 1:1, 2:1 and 4:1 are denoted as Al_{13}-CN-1, Al_{13}-CN-2, Al_{13}-CN-3 and Al_{13}-CN-4, respectively). Error bars in **a** represent the standard deviations of three independent measurements.

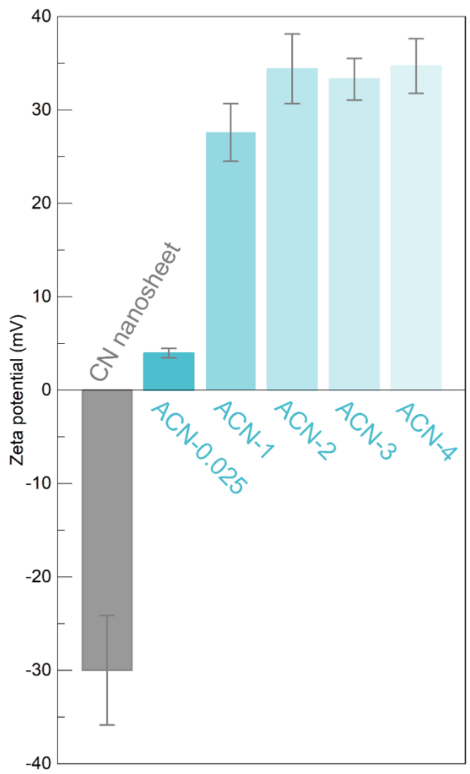

Supplementary Fig. 7 | Zeta potentials of CN nanosheets and Al_{30}-CN. Error bars represent the standard deviations of three independent measurements.

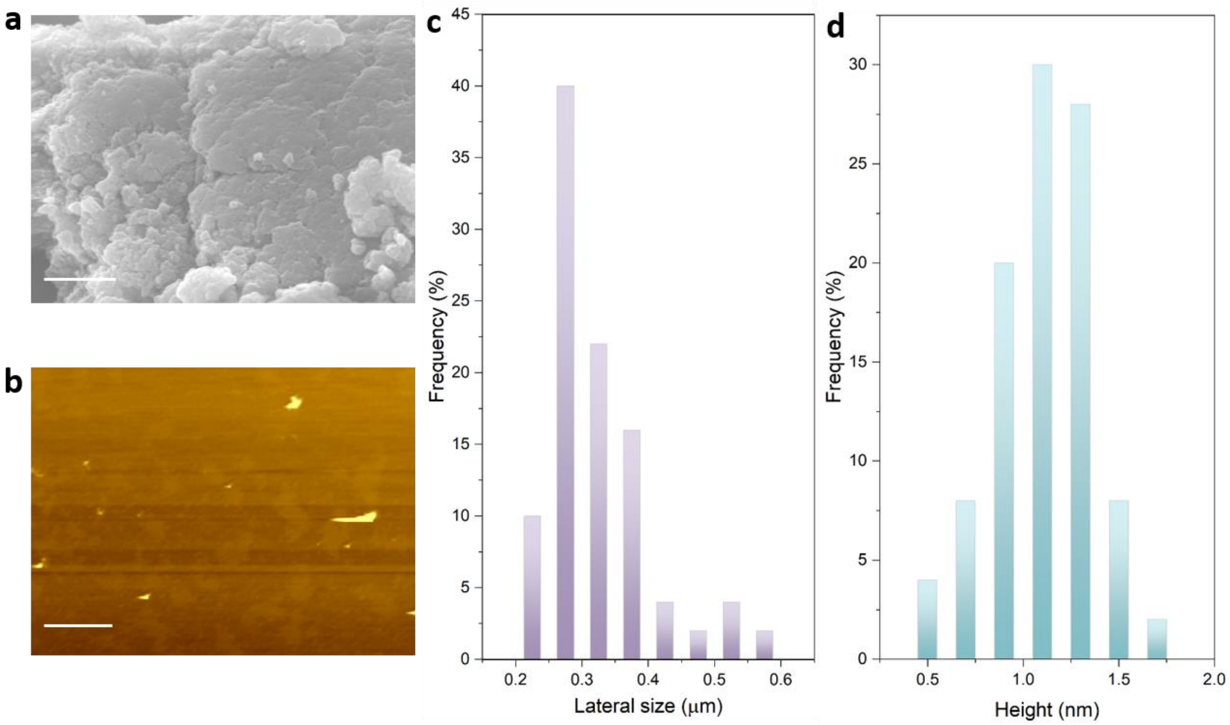

Supplementary Fig. 8 | Morphology and statistical analyses of CN samples. a, SEM image of pristine bulk CN (scale bar: 1 μm). **b-d,** AFM image (**b**) of exfoliated CN nanosheet (scale bar: 500 nm), (**c**) lateral size and (**d**) height distribution profiles.

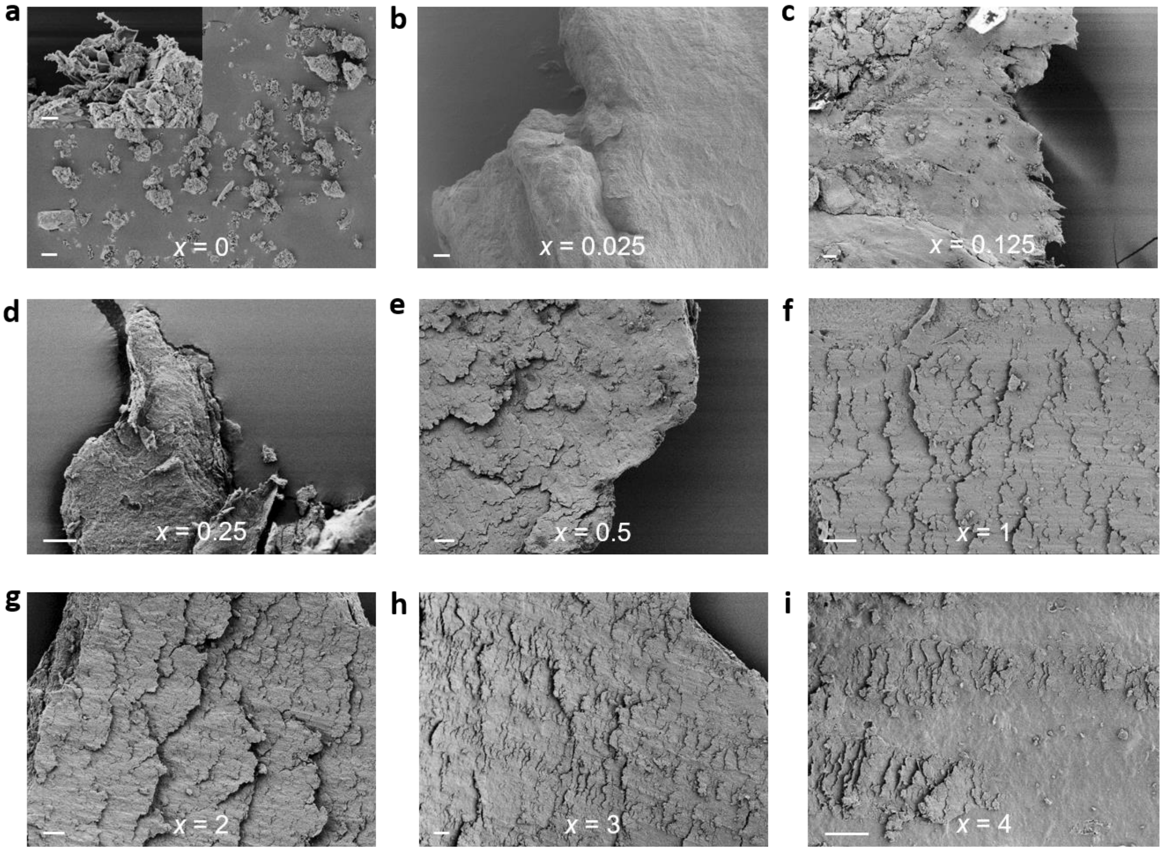

Supplementary Fig. 9 | SEM images of ACN powders. a, CN nanosheets after aging (scale bar: 10 µm) and the magnified image (scale bar: 2 µm). **b-i,** ACN powders with increasing Al_{30} amount (*x*: weight ratio of $[Al_{30}]/[CN]$, scale bar: 10 µm).

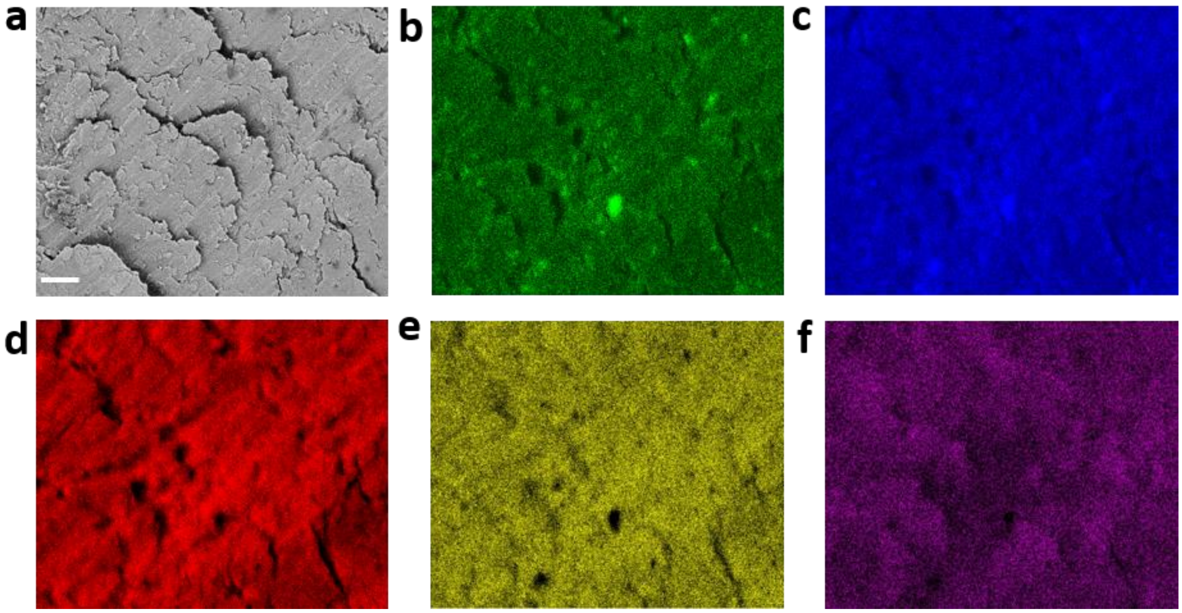

Supplementary Fig. 10 | EDX mapping result of ACN-3 powder. a, SEM image of ACN-3 powder (scale bar: 2 μm). **b-f,** Elemental mapping results. **b,** C. **c,** N. **d,** O. **e,** Al. **f,** Cl.

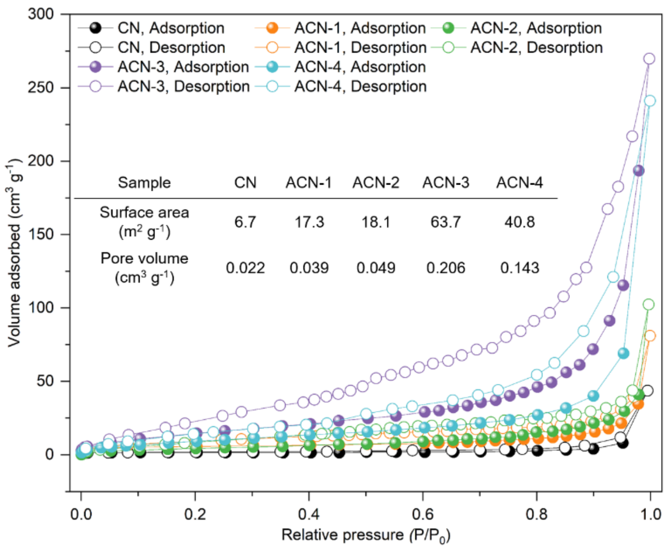

Supplementary Fig. 11 | Nitrogen sorption isotherms of CN and ACN samples recorded at 77 K. Inset shows the surface area and pore volume.

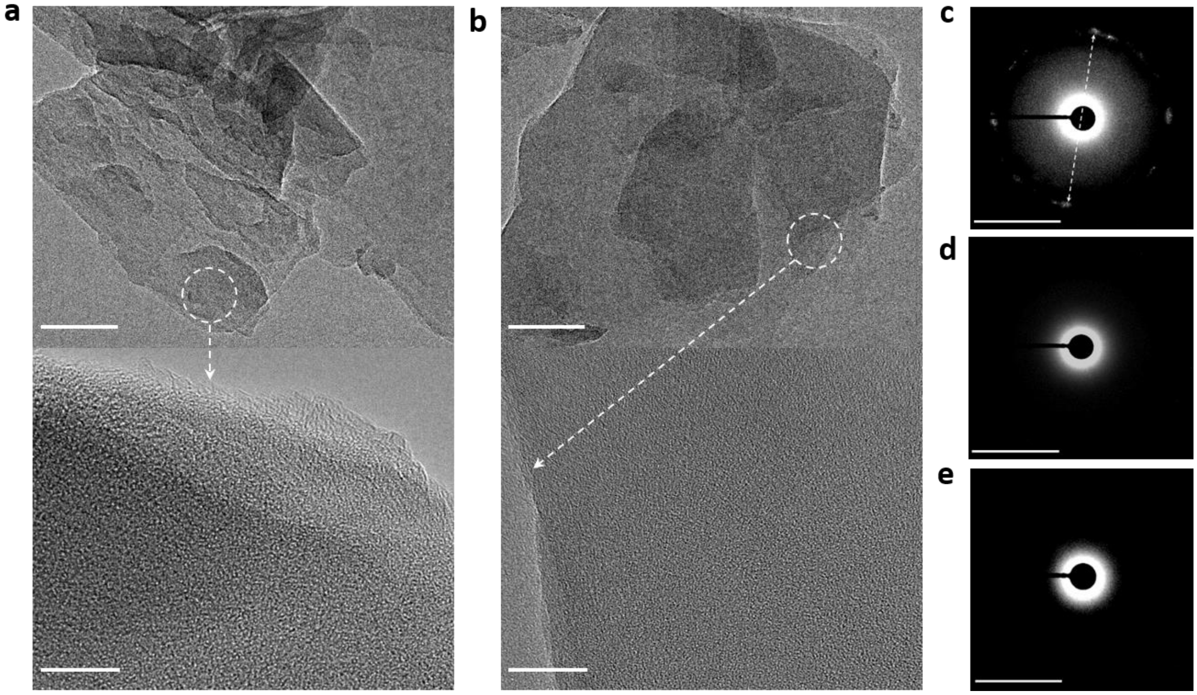

Supplementary Fig. 12│TEM and selected-area electron diffraction (SAED) images of nanosheets. a, CN nanosheets, **b,** Al_{13}-CN nanosheets, **c-e,** SAED images of ACN (**c**), CN (**d**) and Al_{13}-CN (**e**). Scale bars: **a, b,** 50 nm (upper panel) and 10 nm (bottom panel), **c-e,** 1 nm^{-1}. The arrow in **c** denotes an interlayer distance of around 9.9 Å.

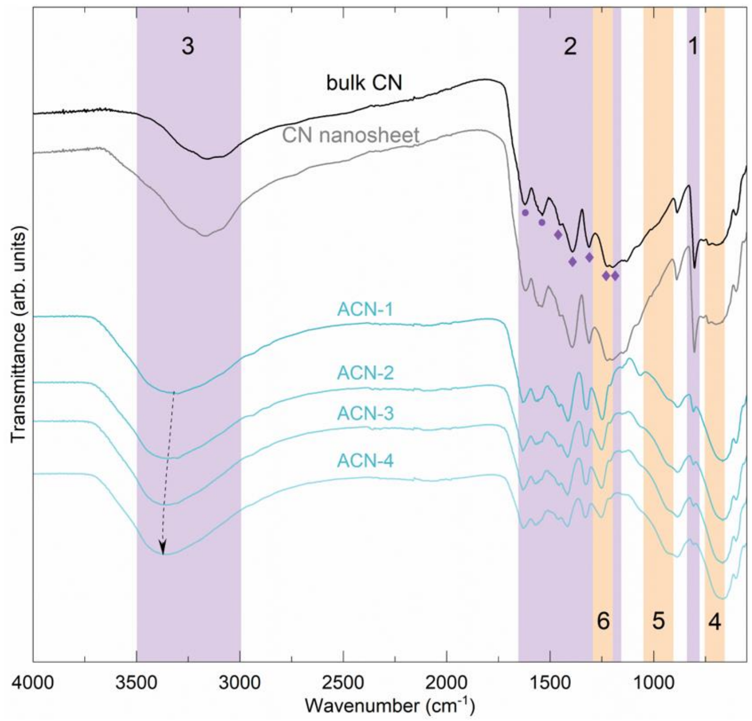

Supplementary Fig. 13 | FT-IR spectra of bulk CN, CN nanosheet and ACN samples. Region 1: 802 cm^{-1}, Region 2: 1200-1700 cm^{-1}, Region 3: 3000-3300 cm^{-1} in CN samples and 3200-3500 cm^{-1} in Al_{30}-CN samples, Region 4: 700-780 cm^{-1}, Region 5: 910-1050 cm^{-1}, Region 6: peak shift from 1224 cm^{-1} to 1250 cm^{-1}. The arrow in Region 3 indicates the gradual red shift with increasing Al_{30} amount. The five rhombic and two circular dots in Region 2 denote stretching vibrations of C-N (1198, 1224, 1312, 1393, and 1452 cm^{-1}) and C=N (1538 cm^{-1} and 1621 cm^{-1}), respectively.

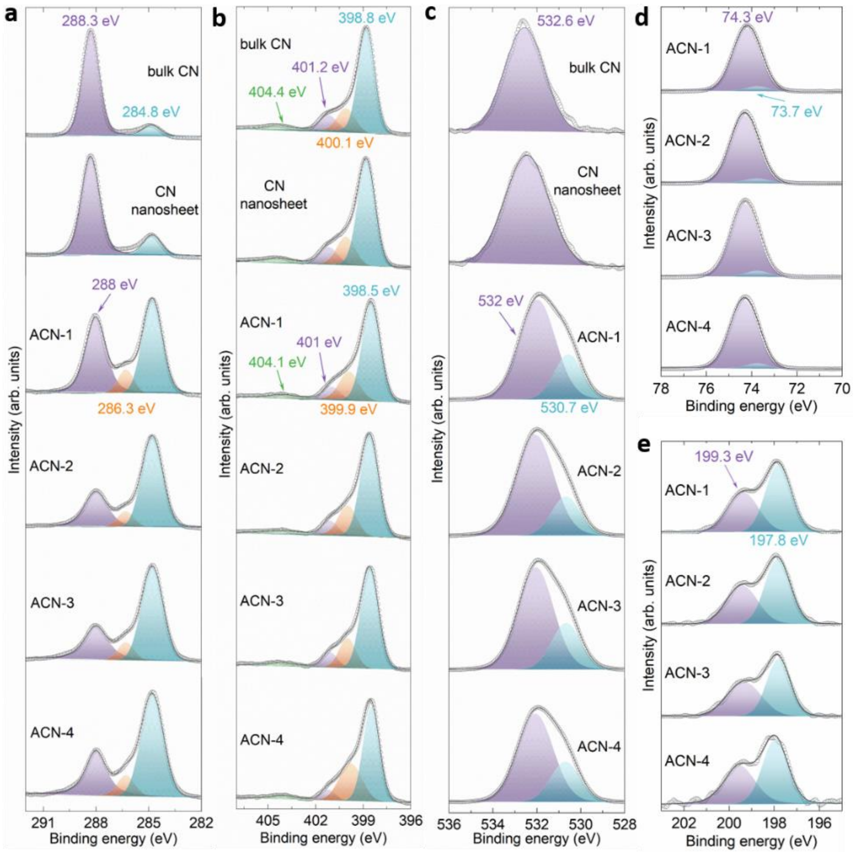

Supplementary Fig. 14 | XPS spectra of CN and ACN samples. a, C 1*s*. **b,** N 1*s*. **c,** O 1*s*. **d,** Al 2*p*. **e,** Cl 2*p*.

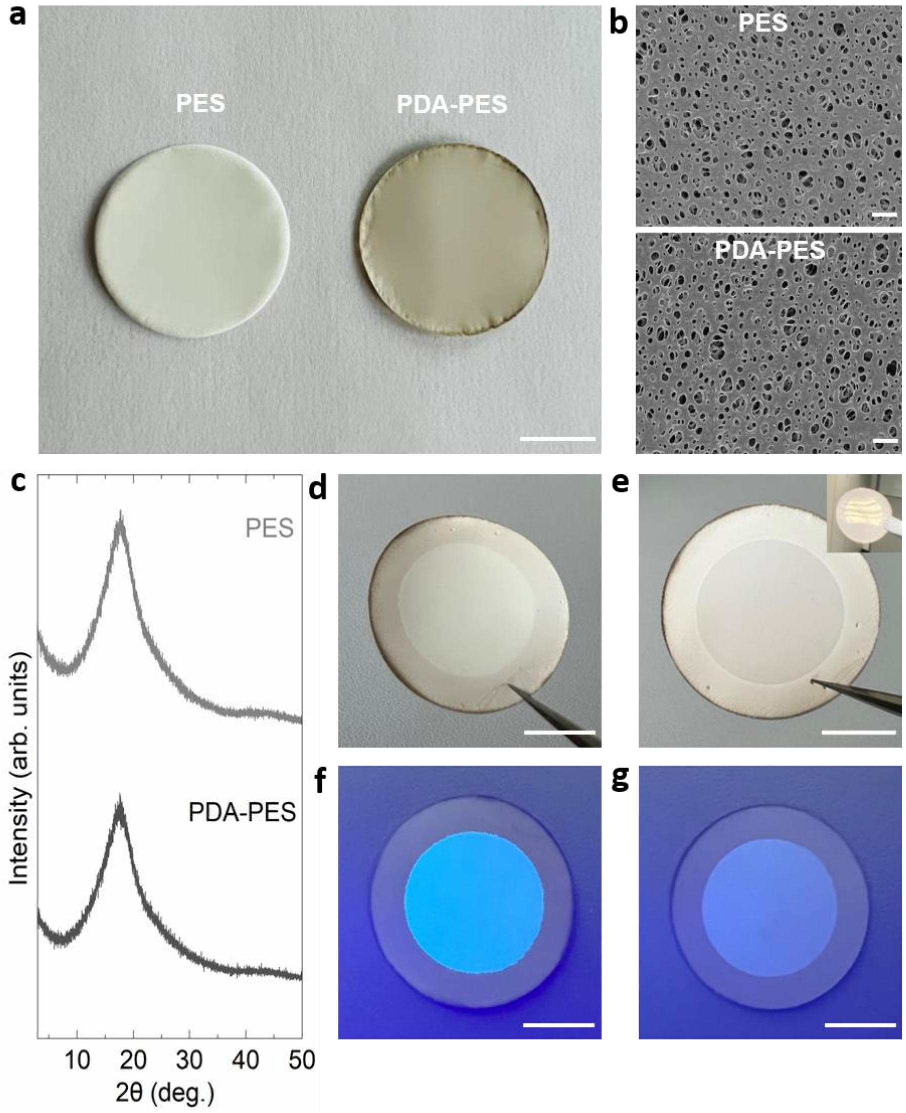

Supplementary Fig. 15 | The filters and membranes used in this work. a, Digital photos (scale bar: 1 cm). **b,** SEM images of bare PES and PDA-PES filters (scale bar: 2 μm). **c,** XRD patterns of PES and PDA-PES filters. **d, e,** Digital photos of CN membrane (**d**) and ACN-3 membrane (**e**) supported by PDA-PES. **f, g,** CN membrane (**f**) and ACN-3 membrane (**g**) under UV lamp (366 nm) for enhanced visuality, scale bars for (**d-g**) are all 1 cm. PDA coating helps improve the affinity and adhesion of our membrane with PES filter, the coating process does not alter the porous structure of PES. For ion permeation and forward osmosis tests, we used the PDA-PES supported membranes; for observation of membrane morphology, we used the free-standing membrane fragments to avoid the interference from filters (see Supplementary Methods section for more details).

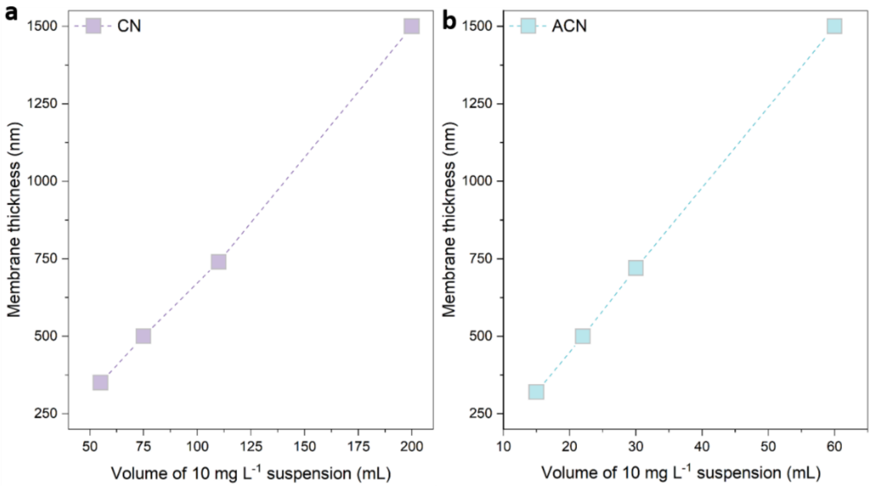

Supplementary Fig. 16 | Variation of membrane thickness as a function of the volume of filtrated suspension. a, CN membrane, **b,** ACN membrane.

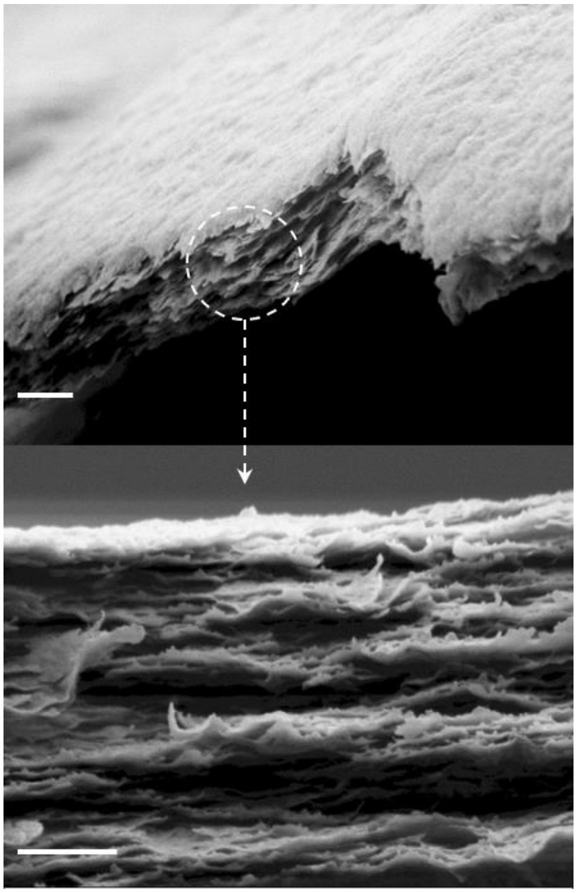

Supplementary Fig. 17 | SEM images of 720 nm-thick ACN-3 membrane. Scale bar: 500 nm (up panel) and 200 nm (bottom panel).

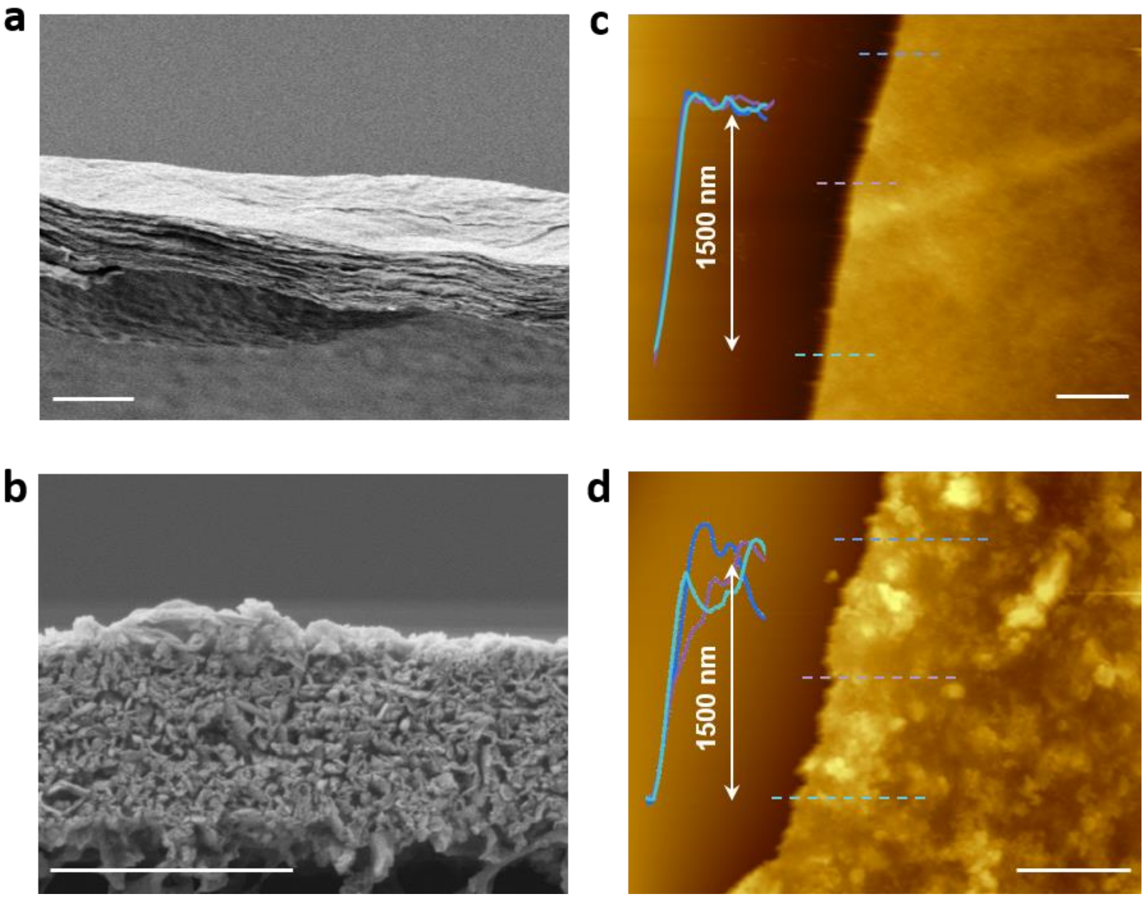

Supplementary Fig. 18 | Microstructural characterization of thicker ACN and CN membranes. a, b, Cross-sectional SEM images of (**a**) ACN-3 and (**b**) CN membrane with thickness of ~1500 nm, scale bar: 2 μm. **c, d,** AFM images of (**c**) ACN-3 (scale bar: 10 μm) and (**d**) CN membranes (scale bar: 10 μm) with corresponding height profiles (inset).

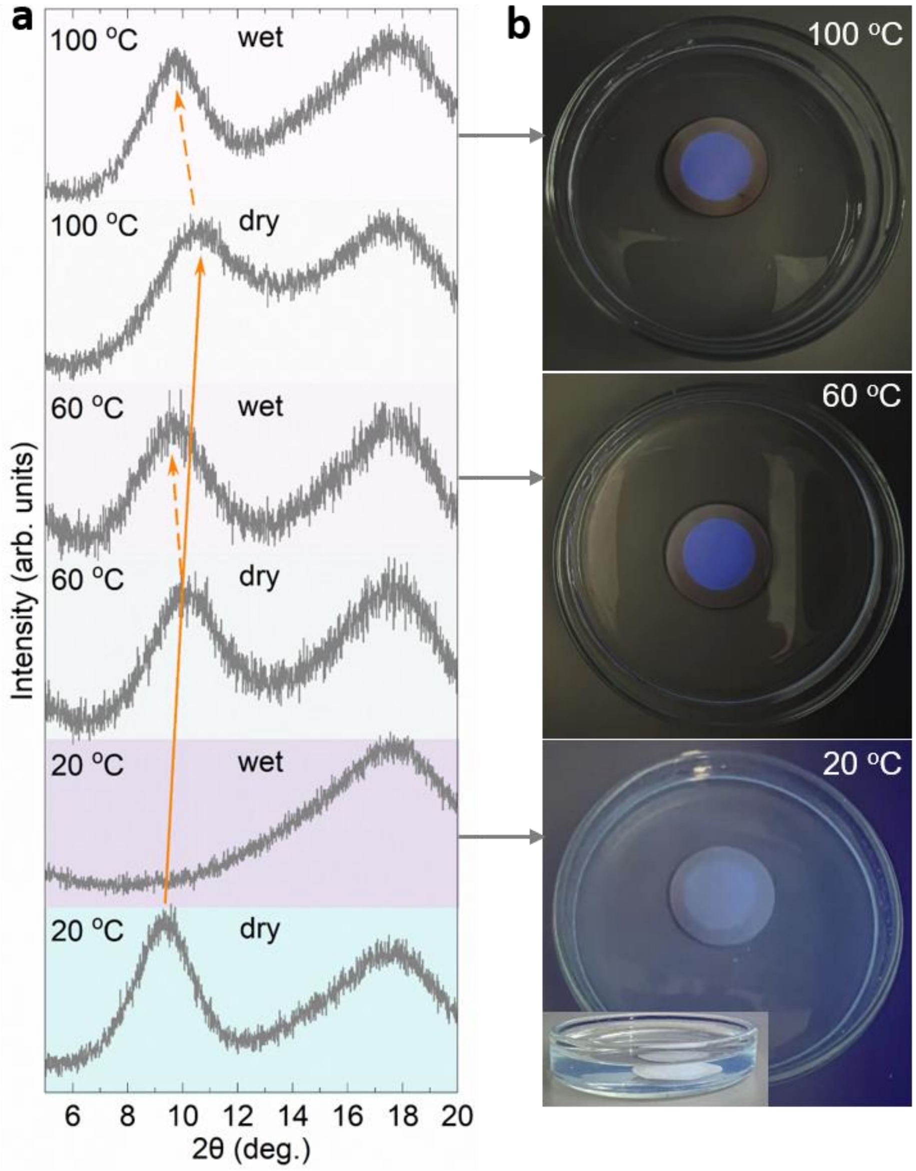

Supplementary Fig. 19 | Membrane stability. a, XRD patterns of ACN-3 membrane dried at different temperatures under vacuum and then immersed in water. **b,** The corresponding digital photos of membranes dried at different temperatures and then immersed in water for 24 h, the digital photos were recorded by irradiating the membranes using a UV lamp (366 nm) to enhance visuality.

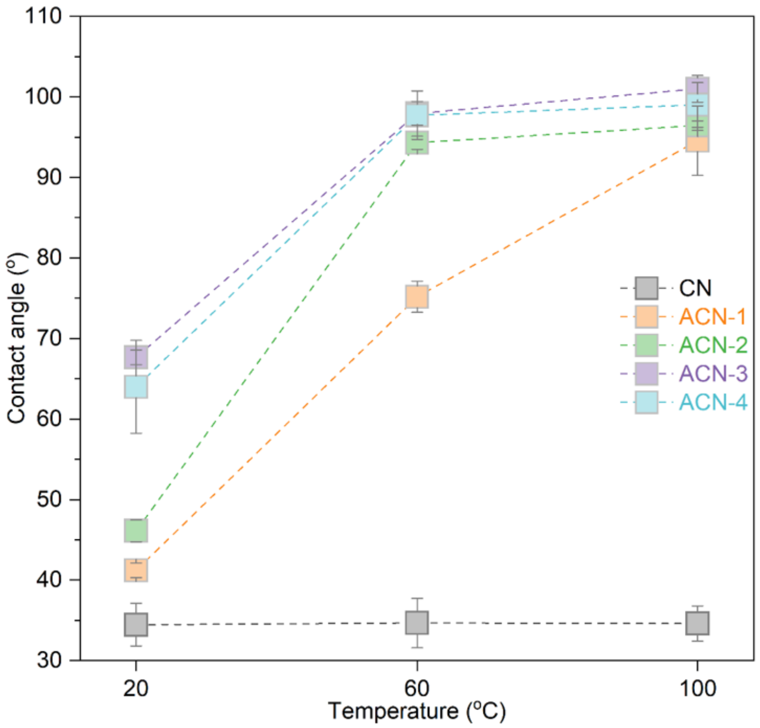

Supplementary Fig. 20│Contact angles of CN and ACN membranes dried at different temperatures under vacuum. Error bars represent the standard deviations of three independent measurements.

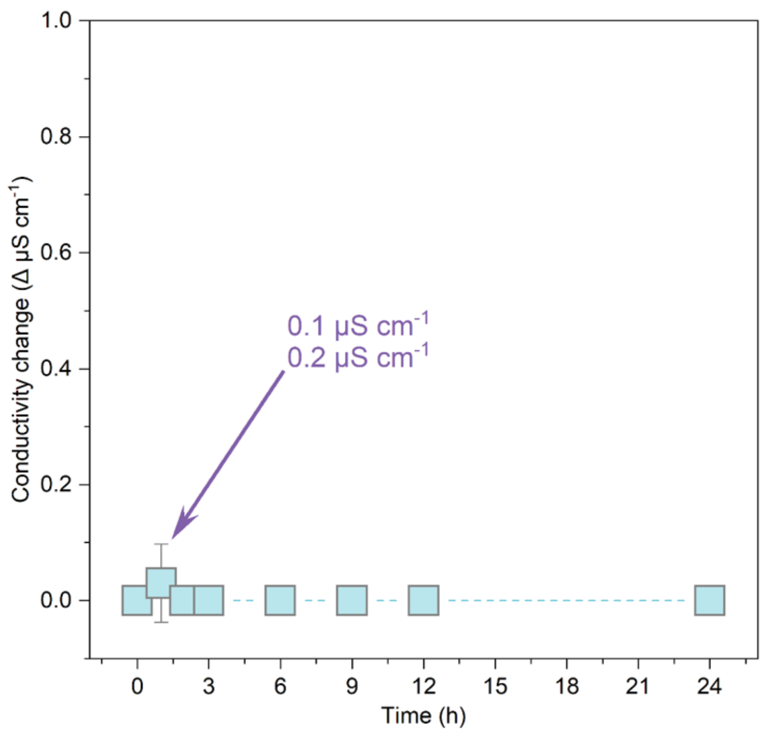

Supplementary Fig. 21 | Conductivity change of ACN-3 membrane as a function of soaking time. The membrane dried at 60 °C under vacuum was soaked in 20 mL H_2O for 24 h under stirring, during which the ionic conductivity of the solution was monitored. Error bars represent the standard deviations of three independent measurements. Among the randomly selected ten membranes from different batches, only two out of them showed subtle conductivity (0.1 and 0.2 μS cm^{-1}) change at the first hour, as marked by the arrow.

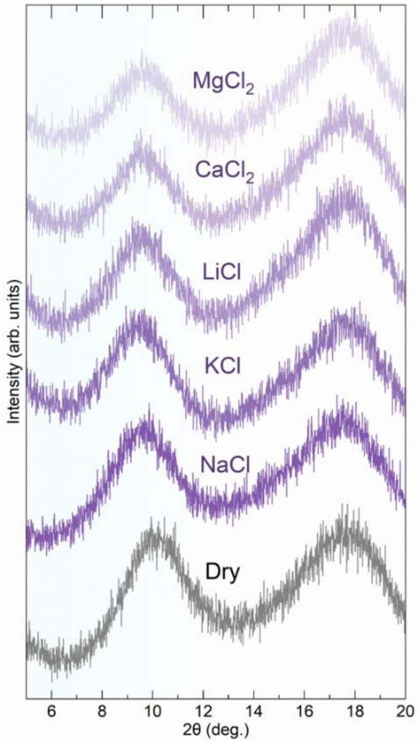

Supplementary Fig. 22 | XRD patterns of ACN-3 membranes immersed in various aqueous salt solutions for 24 h.

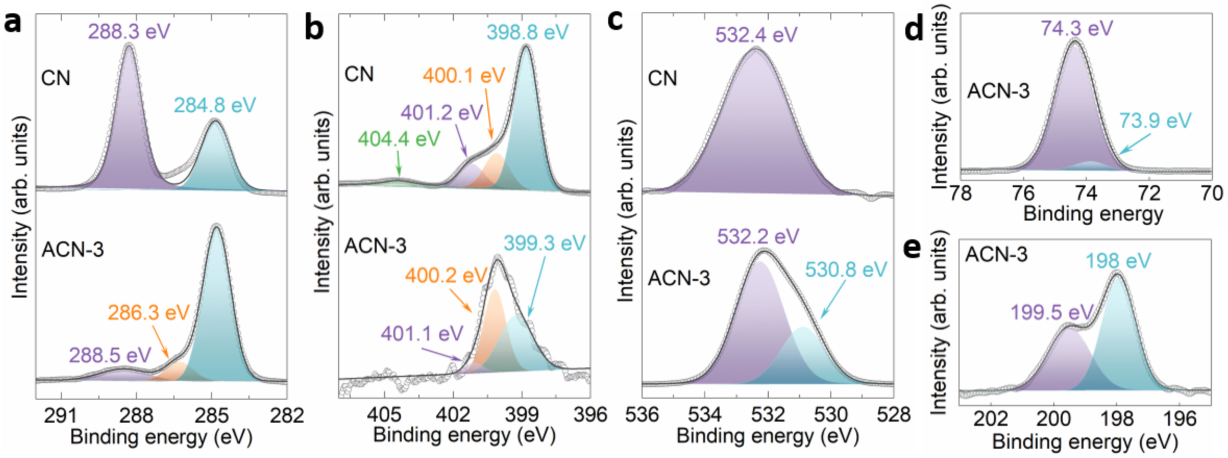

Supplementary Fig. 23 | XPS spectra of CN and ACN-3 membranes. a, C 1*s*. **b,** N 1*s*. **c,** O 1*s*. **d,** Al 2*p*. **e,** Cl 2*p*.

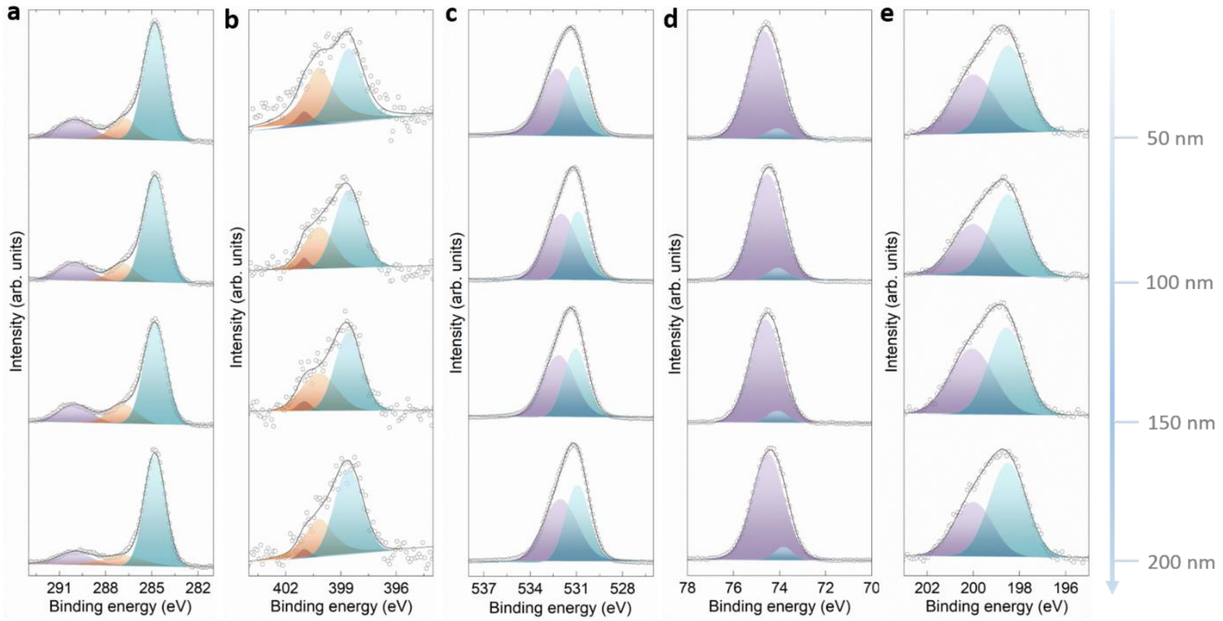

Supplementary Fig. 24 | Etching depth XPS profile of 500 nm-thick ACN-3 membrane. a, C 1*s*. **b,** N 1*s*. **c,** O 1*s*. **d,** Al 2*p*. **e,** Cl 2*p*.

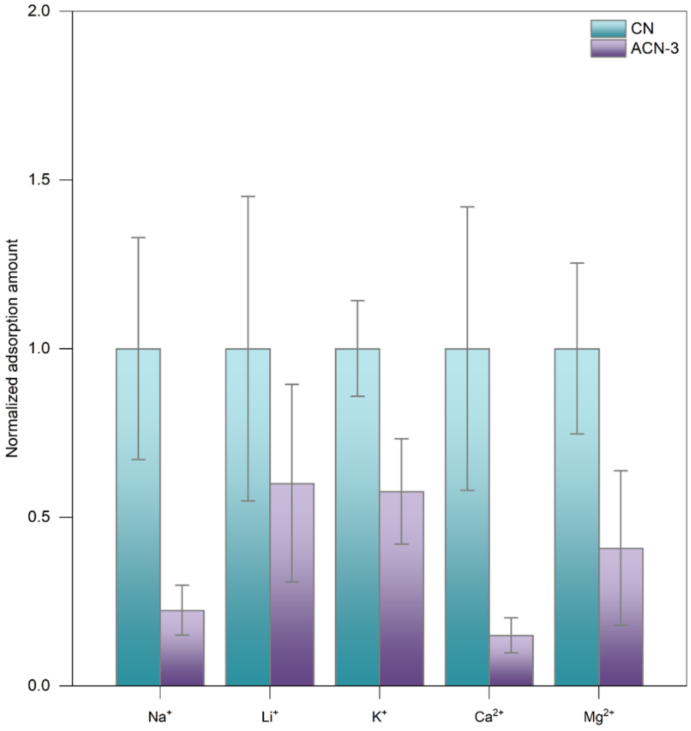

Supplementary Fig. 25 | Ion adsorption of CN and ACN-3 membranes. The membranes were soaked in 0.2 M salt solutions for 24 h, the concentration difference before and after soaking was determined by ICP-OES. Error bars represent the standard deviations of three independent measurements.

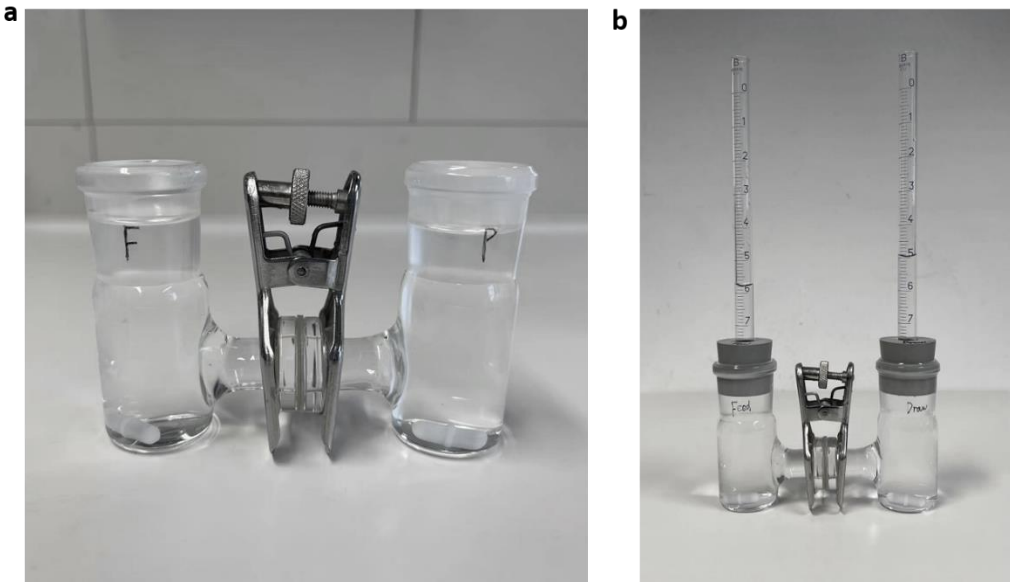

Supplementary Fig. 26 │ Customized H-shaped cells for permeation and separation. a, Ion permeation test (left: feed side, right: permeate side). **b,** Forward osmosis test (left: feed side, right: draw side).

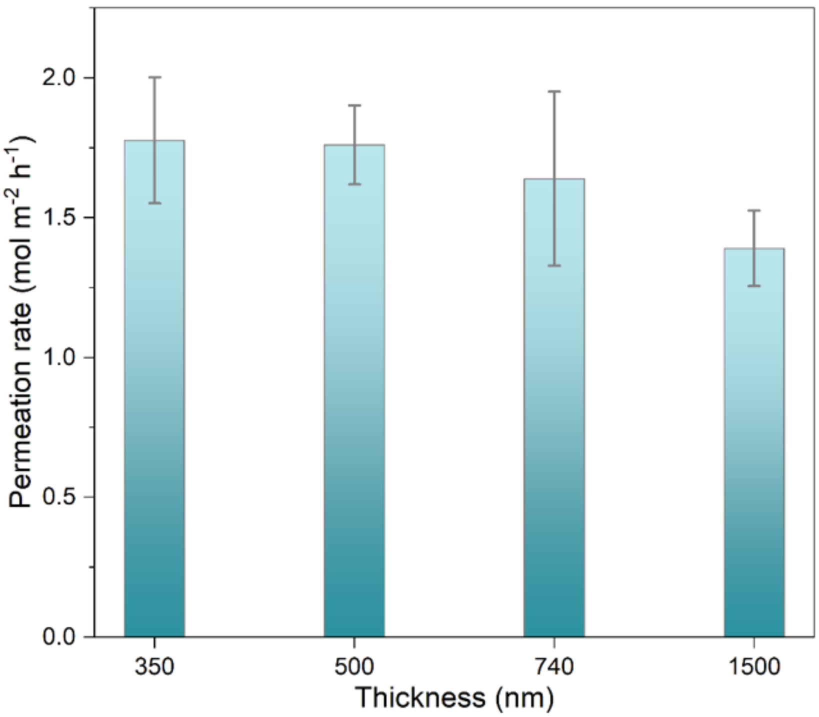

Supplementary Fig. 27 | Na$^+$ permeation rate of CN membrane with different thicknesses. Error bars represent the standard deviations of three independent measurements.

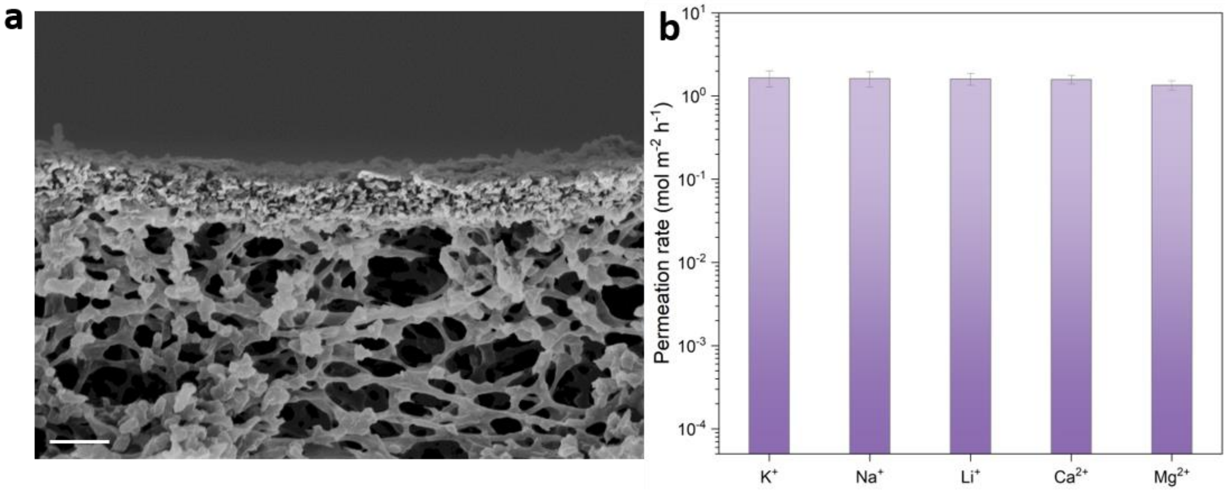

Supplementary Fig. 28 │ Cross-sectional SEM image (a) and ion permeation behavior (b) of Al_{13}-CN membrane (thickness: ~750 nm). Scare bar in **a**: 1 µm. Error bars in **b** represent the standard deviations of three independent measurements.

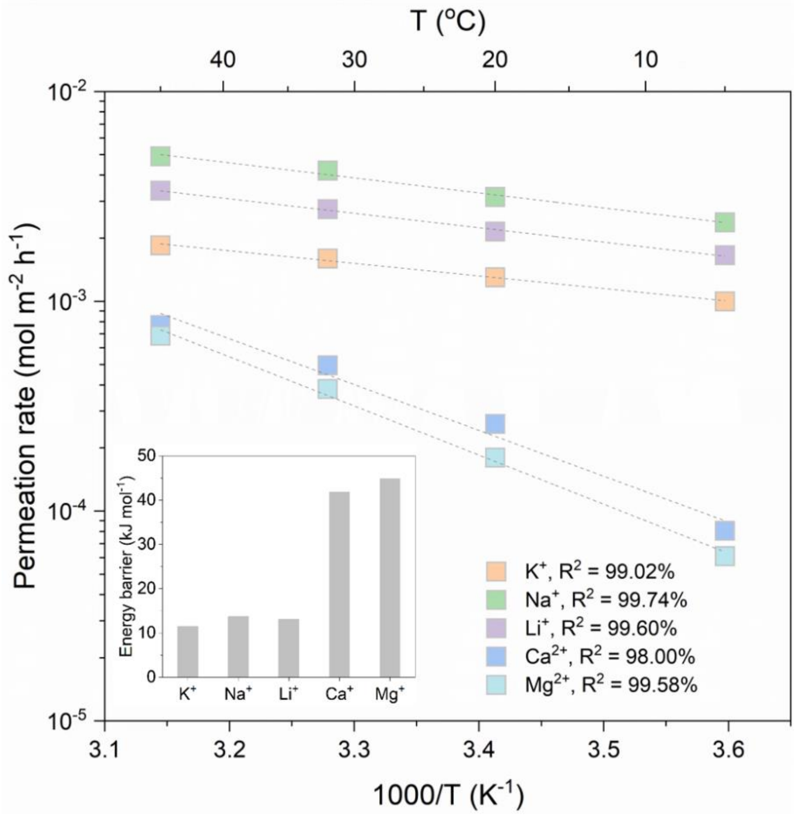

Supplementary Fig. 29 | Temperature dependent ion permeation of ACN-3 membranes. Dotted lines are best fits to the Arrhenius behavior. Inset: energy barriers for various ions.

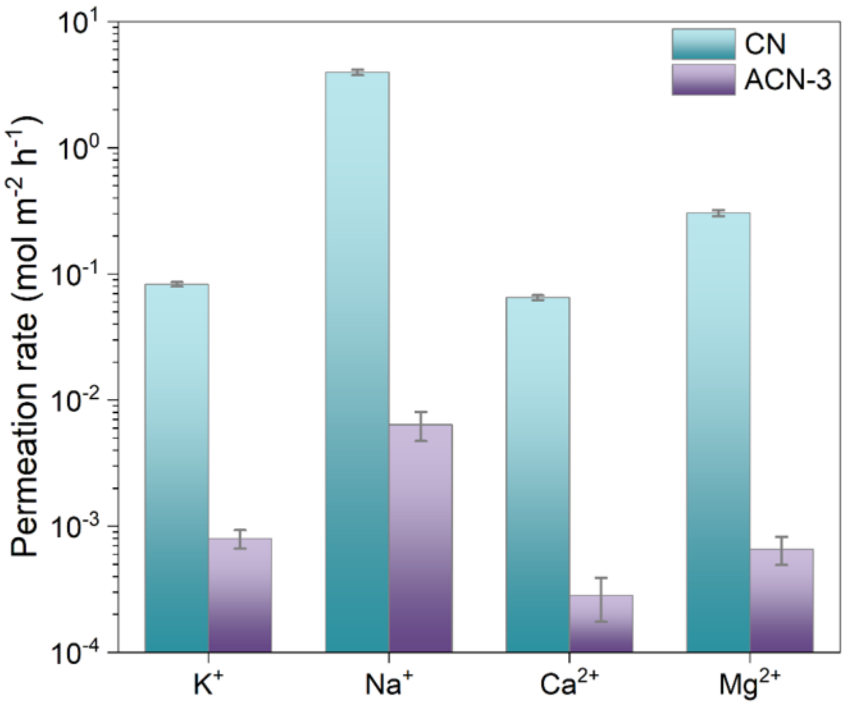

Supplementary Fig. 30 | Mixed ion permeation rates of CN and ACN-3 membranes using synthetic seawater as feed solution. The seawater comprises 9.3×10^{-3} M KCl, 4.2 M NaCl, 2.9×10^{-2} M Na_2SO_4, 1.1×10^{-2} M $CaCl_2$ and 5.6×10^{-2} $MgCl_2$. Error bars represent the standard deviations of three independent measurements.

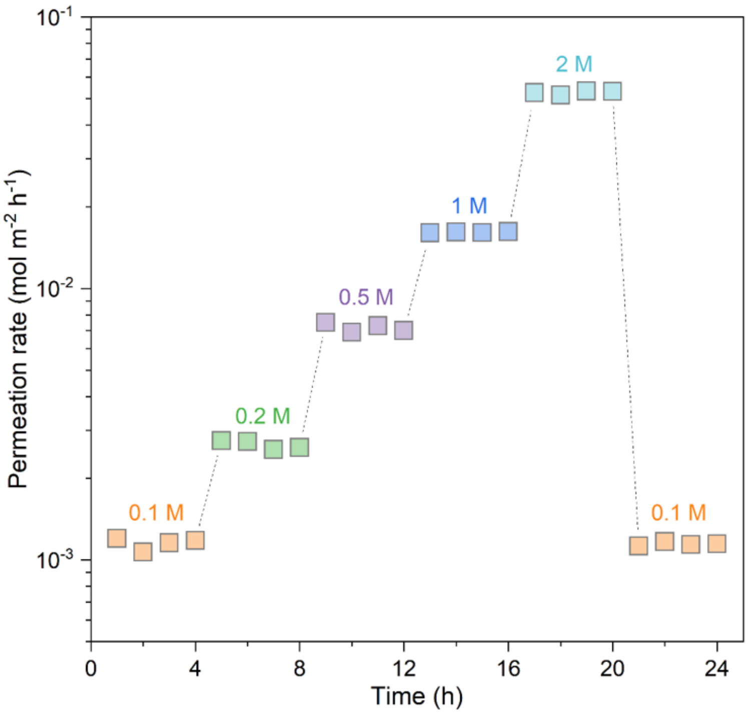

Supplementary Fig. 31 | Na^+ permeation rates of ACN-3 membrane with different initial NaCl concentrations as feed solutions. After permeation for 20 h, both the feed and permeate compartments were thoroughly washed with water, which were then filled with 0.1 M NaCl and water, respectively.

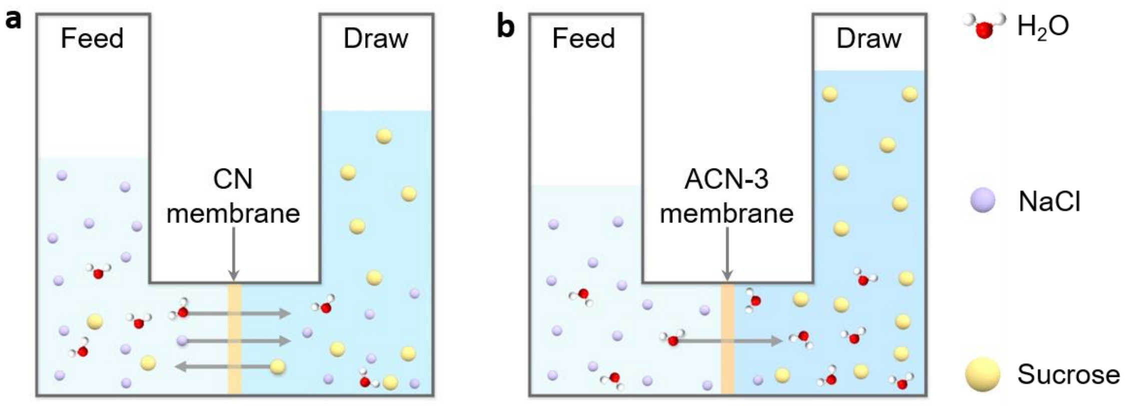

Supplementary Fig. 32 | Different transportation behavior in (a) CN and (b) ACN-3 membrane in forward osmosis. a, In pristine CN membrane, the concomitant transport of H_2O, NaCl and sucrose would decrease the osmotic pressure, leading to the decreased water flux. **b,** In pillared ACN-3 membrane, only H_2O molecules and small amount of NaCl are allowed to permeate into draw side; in this way, the osmotic pressure maintains at higher level and contributes to steady water flux.

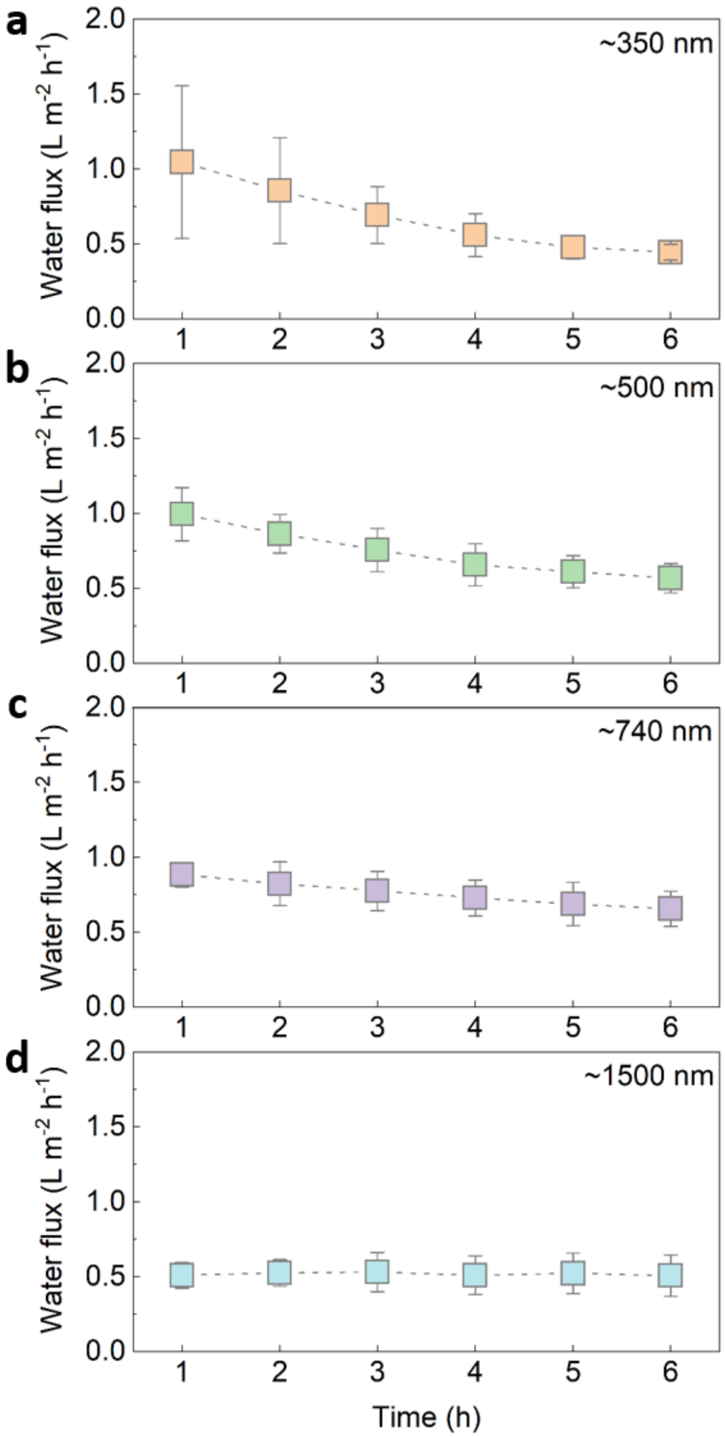

Supplementary Fig. 33│Water flux of CN membrane with different thickness in forward osmosis. a, 300 nm. **b,** 500 nm. **c,** 720 nm. **d,** 1500 nm. Error bars in **a-d** represent the standard deviations of three independent measurements. The time-dependent water flow is evident in thin membranes and gets mitigated when the thickness increases, but coupled with the decrease of water flux.

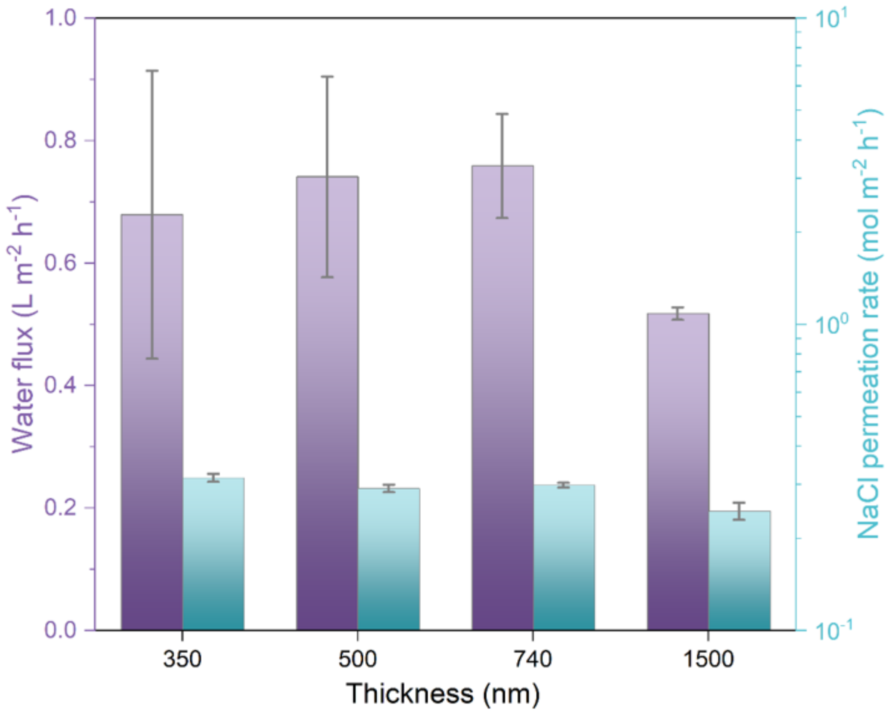

Supplementary Fig. 34 | Water flux and NaCl permeation rate as a function of CN membrane thickness. Error bars represent the standard deviations of three independent measurements.

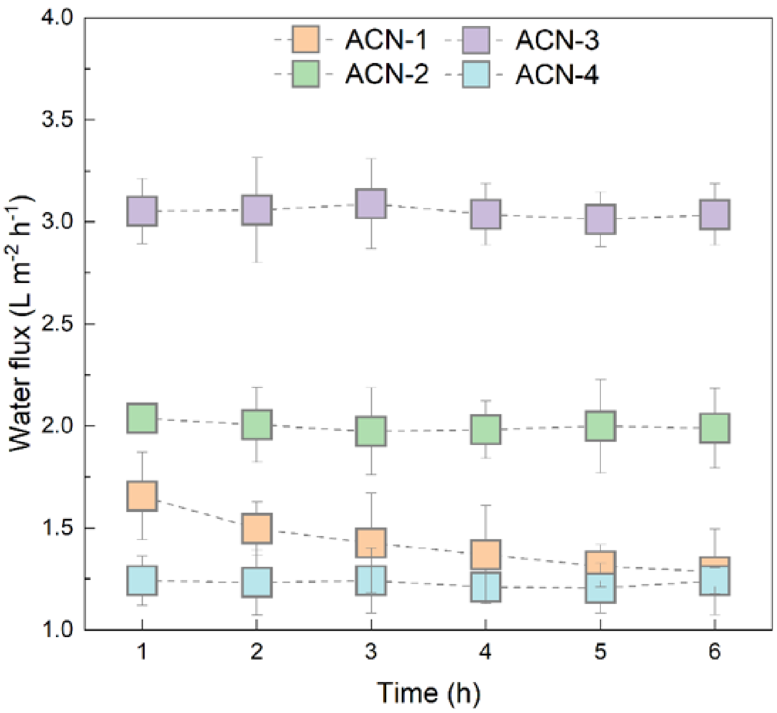

Supplementary Fig. 35│Water flux variation of 500-nm thick ACN membranes with prolonging permeation time in forward osmosis. Error bars represent the standard deviations of three independent measurements. Although being less evident relative to that of CN membrane (Supplementary Fig. 33), the time-dependent water flow remains in ACN-1 membrane, due to the less regular sub-nanochannel with lower Al_{30} amount (Fig. 2a in main text). On the other hand, overloading of Al_{30} may block the transport passage, which explains the decreased water flux, although the permeation rate of NaCl is slightly decreased (Fig. 4c in main text). Overall, the ACN-3 membrane combines high water flux and salt rejection.

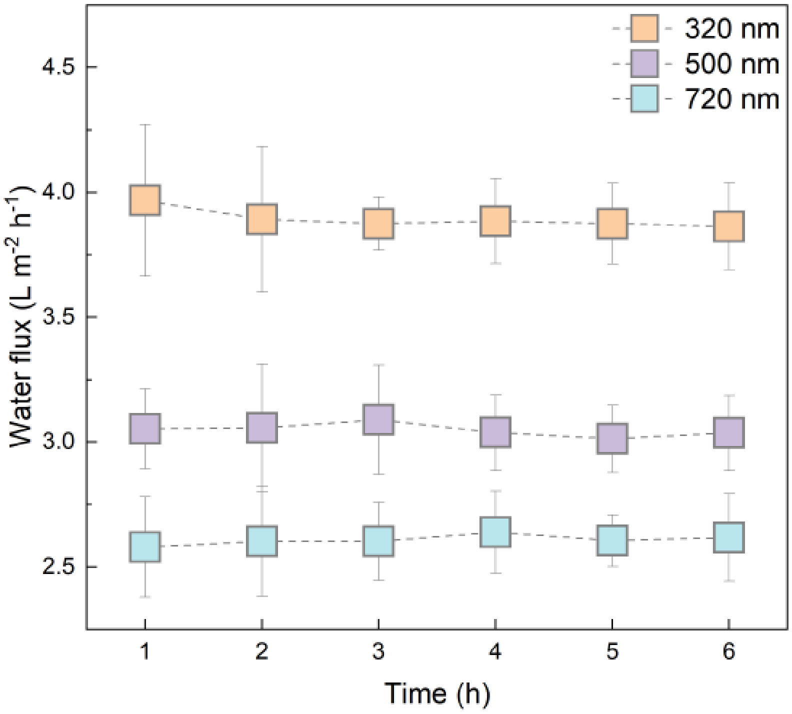

Supplementary Fig. 36 | Variation of water flux of ACN-3 membranes with different thickness as a function of permeation time. Error bars represent the standard deviations of three independent measurements.

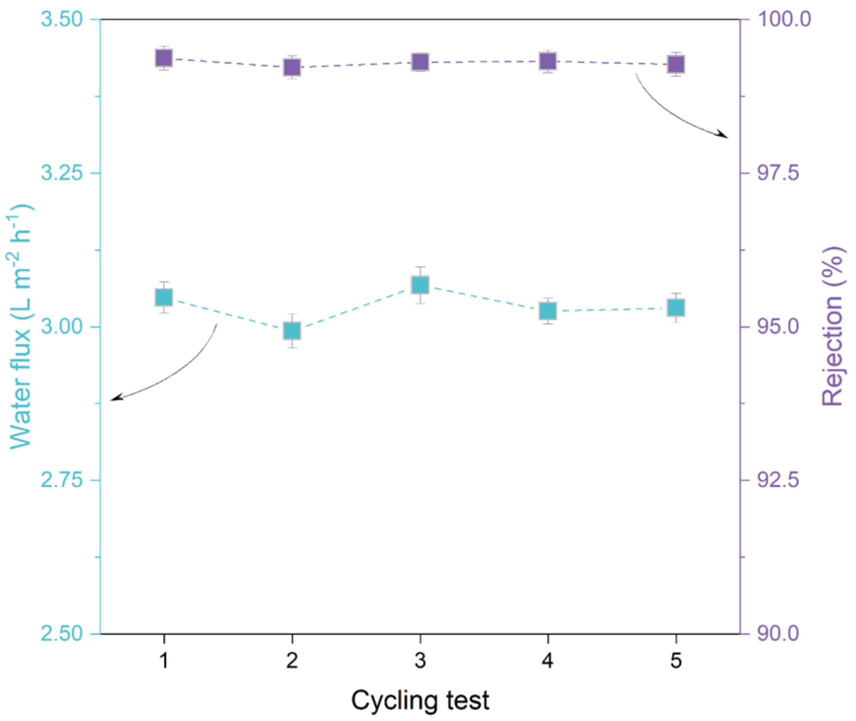

Supplementary Fig. 37 | Cycling test of 500 nm-thick ACN-3 membrane upon immersion treatment. Prior to next-cycle test, the membrane was immersed in H_2O for 12 h. Error bars represent the standard deviations of three independent measurements.

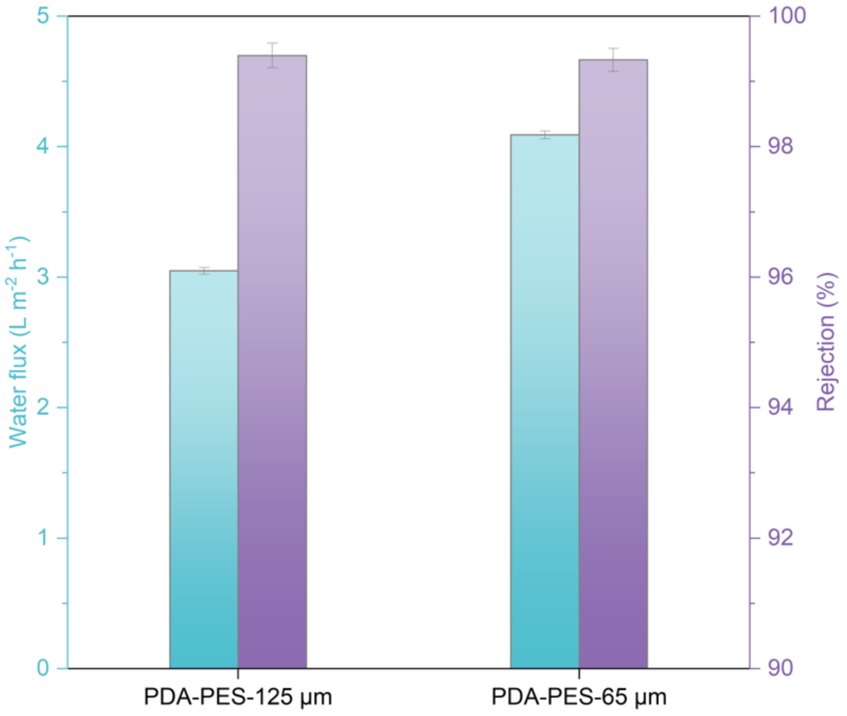

Supplementary Fig. 38 | Water flux and salt rejection of 500 nm-thick ACN-3 membrane supported by PDA-PES filter with different thicknesses. Error bars represent the standard deviations of three independent measurements.

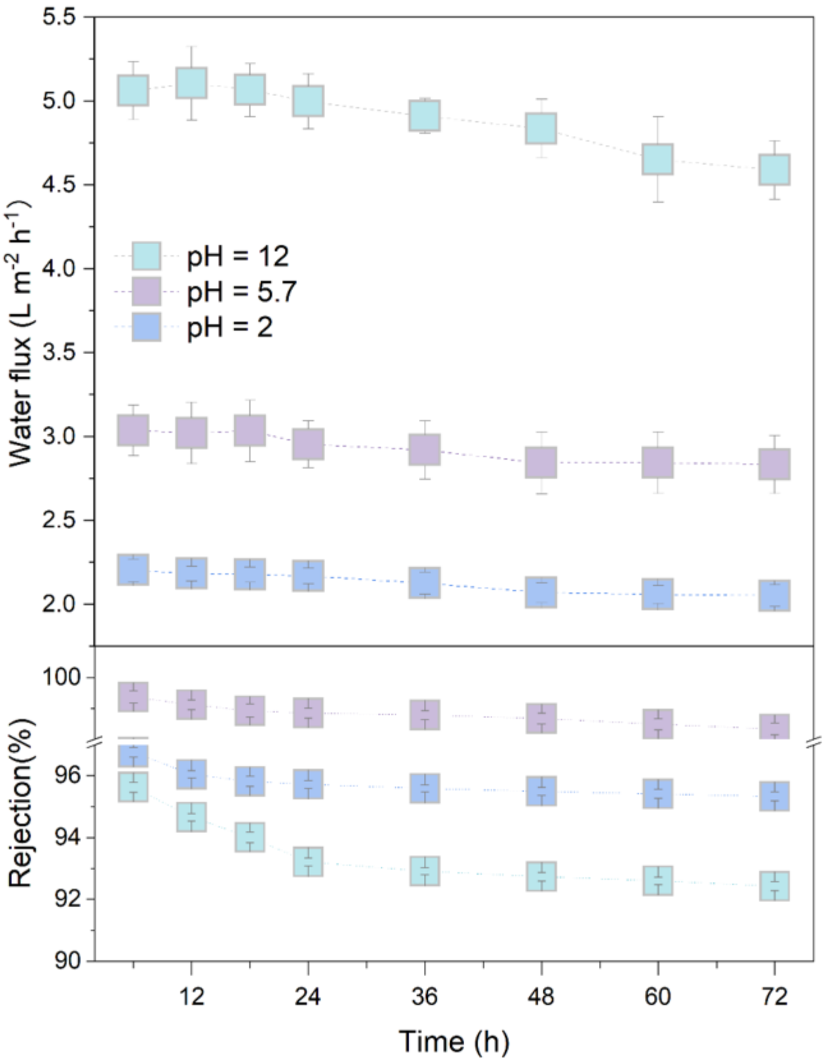

Supplementary Fig. 39│Long-term stability test of ACN-3 membrane operated in acidic, neutral and alkaline solutions. Water flux and salt rejection are plotted as a function of permeation time up to 72 hours. Error bars represent the standard deviations of three independent measurements.

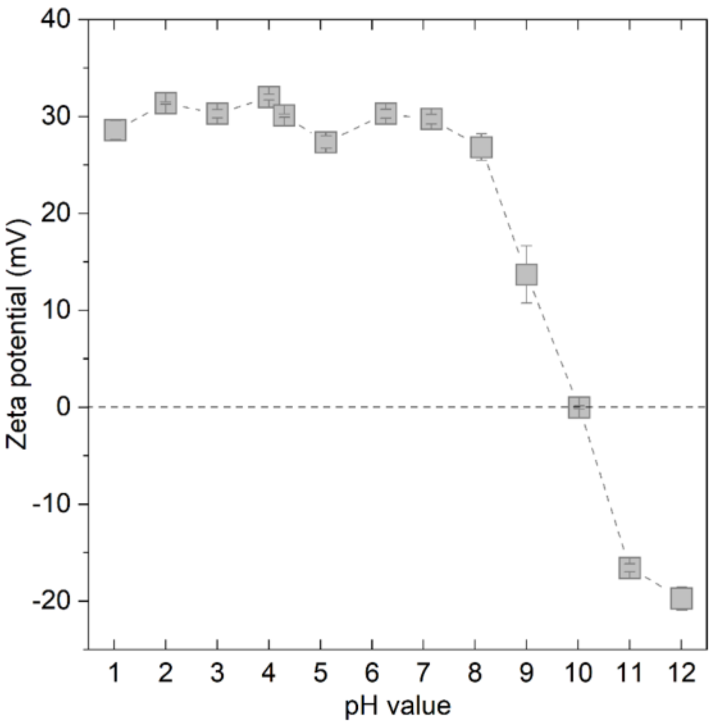

Supplementary Fig. 40 | Zeta potential variation of ACN-3 composite at different pH values. The isoelectric point occurs at pH = 10.0. Error bars represent the standard deviations of three independent measurements.

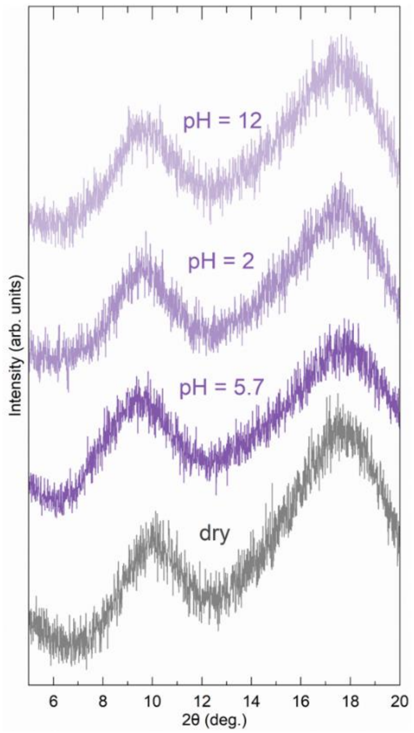

Supplementary Fig. 41│XRD patterns of ACN-3 membranes immersed in water environments with varied pH values for 24 h.

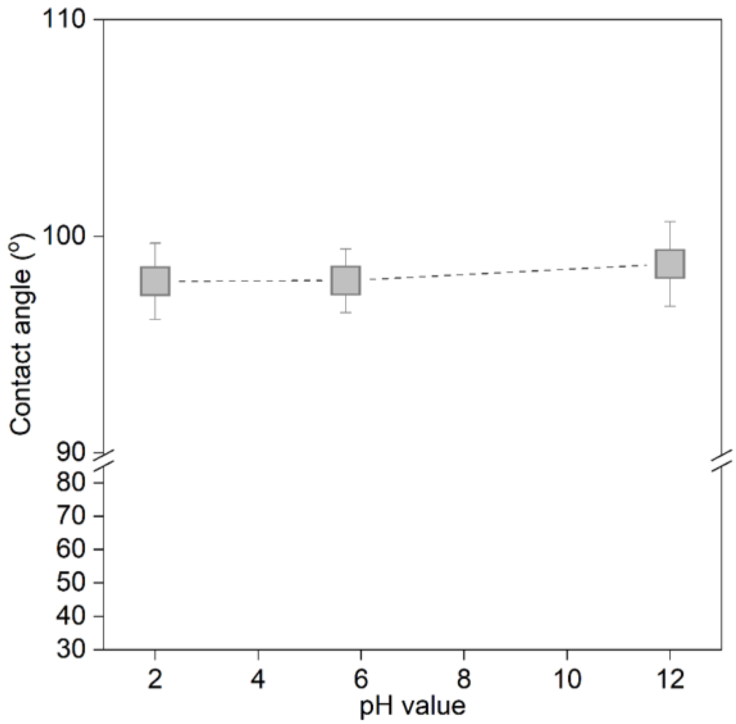

Supplementary Fig. 42 | Water contact angles of ACN-3 membranes at different pH values. Error bars represent the standard deviations of three independent measurements.

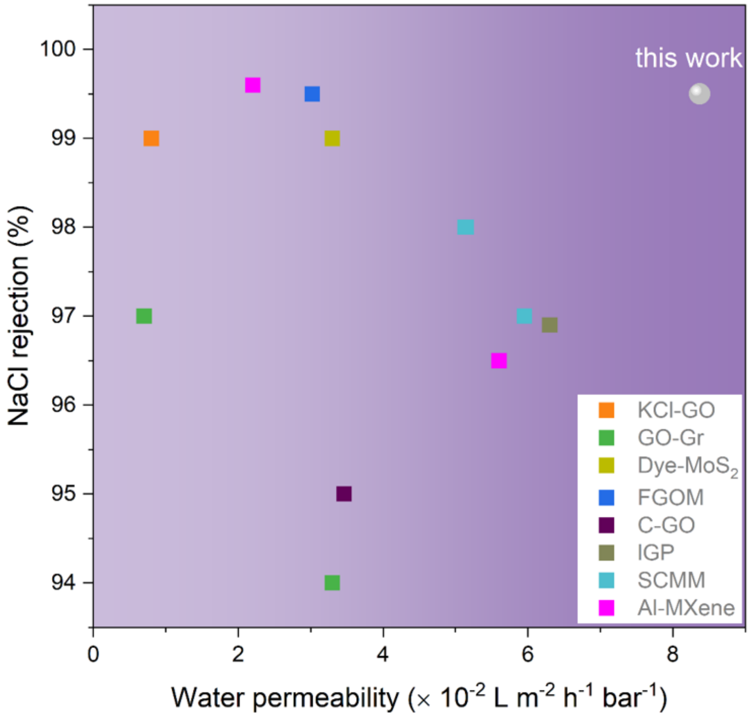

Supplementary Fig. 43 | Water permeability and NaCl rejection performance of 2D membranes in forward osmosis. The salt rejection values higher than 94% in Supplementary Table 3 were used for comparison, the data of "this work" was from Supplementary Fig. 38.

Supplementary Table 1 | Chemical environment variation in CN and ACN samples.

Sample	CN nanosheet	ACN-1	ACN-2	ACN-3	ACN-4
(C-N=C)/(C-NH_x)	6.56	8.20	9.21	9.37	10.78
(C-$(N)_3$)/(C-NH_x)	1.71	2.21	2.69	2.93	5.79
Al_{30}/CN*	0	0.29	0.80	1.00	0.62

*: Al_{30}/CN denotes the ratio of Al_{30} to the tri-*s*-triazine unit in the membrane, which was determined by Argon-ion sputtered XPS depth profiles in Supplementary Fig. 24. One can see that the ratio increases with the increment of Al_{30} amount. However, the ratio is decreased in ACN-4, suggesting that Al_{30} overloading is detrimental to the formation of lamellar structure in ACN composite. This is also reflected by the SEM images of ACN powders, in which the lamellar texture in ACN-4 sample turns less identifiable.

Supplementary Table 2 | Diameter of effective and hydrated ions and their hydration free energy.

Ion	K^+	Na^+	Li^+	Ca^{2+}	Mg^{2+}
Effective diameter (Å)*	2.76	2.04	1.52	2.00	1.44
Hydrated diameter (Å)*	6.62	7.16	7.64	8.24	8.56
Hydration free energy (kJ mol^{-1})*	-321	-405	-515	-1592	-1922

*: The relevant data are acquired from previous studies[23,24].

Supplementary Table 3 | Comparison of water desalination performance of 2D membranes driven by osmotic pressure.

Membrane material	Thickness (nm)	Draw solution	Water flux (L $m^{-2}h^{-1}$)	Water permeability (L m^{-2} h^{-1} bar^{-1})	Salt Rejection (%)	Ref.
**ACN**	320 (pH = 12)	2 M sucrose	5.9	0.120	95.5	**This work**
	320 (pH = 5.7)		3.9	0.080	99.2	
	320 (pH = 2)		2.3	0.047	96.2	
	500 (pH = 12)		5.1	0.104	96.1	
	500 (pH = 5.7)		3.0	0.061	99.4	
	500 (pH = 2)		2.2	0.045	97.3	
	720 (pH = 12)		4.7	0.096	96.3	
	720 (pH = 5.7)		2.6	0.053	99.5	
	720 (pH = 2)		2.0	0.041	97.6	
Dye-functionalized MoS_2	5000	3 M sucrose	2.5	0.033	99	25
MoS_2	1000	1 M NaCl	0.11	0.0022	/	26
KCl-GO	750	0.25 M NaCl	0.1	0.008	99	23
	550		0.23	0.0184	/	
	280		0.36	0.0288	/	
GO	1000	1 M sucrose	0.2	0.008	/	27
GO/Gr	1000	3 M sucrose	2.5	0.033	94	24
	5000		0.5	0.007	97	
FGOM	200	1 M sucrose	0.74	0.03022	99.5	28
C-GO	5300	3 M sucrose	2.49	0.0346	95	29
lGP	3000	3 M sucrose	4.73	0.063	96.9	30
SCMM	500 (SCMM-80)	0.2 M sucrose	0.29	0.0595	97	31
	500 (SCMM-120)		0.25	0.0513	98	
	500 (SCMM-180)		0.25	0.0515	98	
Al-intercalated MXene	340	2 M sucrose	8.5	0.17	89.5	32
	580		4.8	0.096	92.3	
	1100		2.8	0.056	96.5	
	2700		1.1	0.022	99.6	

Supplementary References

1. Wu, Z. *et al*. Transparent, conductive carbon nanotube films. *Science* **305**, 1273-1276 (2004).

2. Zhang, X. *et al*. Enhanced photoresponsive ultrathin graphitic-phase C_3N_4 nanosheets for bioimaging. *J. Am. Chem. Soc.* **135**, 18-21 (2013).

3. Ong, W. J. *et al*. Graphitic carbon nitride (g-C_3N_4)-based photocatalysts for artificial photosynthesis and environmental remediation: are we a step closer to achieving sustainability? *Chem. Rev.* **116**, 7159-7329 (2016).

4. Banerjee, T., Podjaski, F., Kröger, J., Biswal, B. P. & Lotsch, B. V. Polymer photocatalysts for solar-to-chemical energy conversion. *Nat. Rev. Mater.* **6**, 168-190 (2021).

5. Teixeira, I. F., Barbosa, E. C. M., Tsang, S. C. E. & Camargo, P. H. C. Carbon nitrides and metal nanoparticles: from controlled synthesis to design principles for improved photocatalysis. *Chem. Soc. Rev.* **47**, 7783-7817 (2018).

6. Abeysinghe, S., Unruh, D. K. & Forbes, T. Z. Surface modification of Al_{30} Keggin-type polyaluminum molecular clusters. *Inorg. Chem.* **52**, 5991-5999 (2013).

7. Rustad, J. R. Molecular dynamics simulation of the titration of polyoxocations in aqueous solution. *Geochim. Cosmochim. Acta* **69**, 4397-4410 (2005).

8. Huang, C. *et al*. Unraveling fundamental active units in carbon nitride for photocatalytic oxidation reactions. *Nat. Commun.* **12**, 320 (2021).

9. Xu, J., Zhang, L., Shi, R. & Zhu, Y. Chemical exfoliation of graphitic carbon nitride for efficient heterogeneous photocatalysis. *J. Mater. Chem. A* **1**, 14766 (2013).

10. Shalom, M., Inal, S., Fettkenhauer, C., Neher, D. & Antonietti, M. Improving carbon nitride photocatalysis by supramolecular preorganization of monomers. *J. Am. Chem. Soc.* **135**, 7118-7121 (2013).

11. Wang, G., Xu, J., Sun, Z. & Zheng, S. Surface functionalization of montmorillonite with chitosan and the role of surface properties on its adsorptive performance: a comparative study on mycotoxins adsorption. *Langmuir* **36**, 2601-2611 (2020).

12. Zeng, Y. *et al*. Irreversible synthesis of an ultrastrong two-dimensional polymeric material. *Nature* **602**, 91-95 (2022).

13. Liu, W. *et al*. Confined synthesis of carbon nitride in a layered host matrix with unprecedented solid-state quantum yield and stability. *Adv. Mater.* **30**, 1704376 (2018).

14. Lin, L., Ou, H., Zhang, Y. & Wang, X. Tri-s-triazine-based crystalline graphitic carbon nitrides for highly efficient hydrogen evolution photocatalysis. *ACS Catal.* **6**, 3921-3931 (2016).

15. Zhou, Z. *et al*. Dissolution and liquid crystals phase of 2D polymeric carbon nitride. *J. Am. Chem. Soc.* **137**, 2179-2182 (2015).

16. Zhang, Y. *et al*. Reversible assembly of graphitic carbon nitride 3D network for highly selective dyes absorption and regeneration. *ACS Nano* **10**, 9036-9043 (2016).

17. Wang, X., Xu, H. & Wang, D. Mechanism of fluoride removal by $AlCl_3$ and Al_{13}: the role of aluminum speciation. *J. Hazard. Mater.* **398**, 122987 (2020).

18. An, G. *et al*. Deprotonation and aggregation of Al_{13} under alkaline titration: a simulating study related to coagulation process. *Water Res.* **203**, 117562 (2021).

19. Song, W. *et al*. Poly(aluminum chloride) shell-desensitized cyclotrimethylenetrinitramine explosive core. *Mater. Chem. Phys.* **276**, 125335 (2022).

20. Yeh, C. N., Raidongia, K., Shao, J., Yang, Q. H. & Huang, J. On the origin of the stability of graphene oxide membranes in water. *Nat. Chem.* **7**, 166-170 (2015).

21. Zhu, J. *et al*. Keggin-Al_{30} pillared montmorillonite. *Micropor. Mesopor. Mat.* **242**, 256-263 (2017).

22. Akitt, J. W. Multinuclear studies of aluminium compounds. *Prog. Nucl. Mag. Res. Sp.* **21**, 1-149 (1989).

23. Chen, L. *et al*. Ion sieving in graphene oxide membranes via cationic control of interlayer spacing. *Nature* **550**, 380-383 (2017).

24. Abraham, J. *et al*. Tunable sieving of ions using graphene oxide membranes. *Nat. Nanotechnol.* **12**, 546-550 (2017).

25. Hirunpinyopas, W. *et al*. Desalination and nanofiltration through functionalized laminar MoS_2 membranes. *ACS Nano* **11**, 11082-11090 (2017).

26. Deng, M., Kwac, K., Li, M., Jung, Y. & Park, H. G. Stability, molecular sieving, and ion diffusion selectivity of a lamellar membrane from two-dimensional molybdenum disulfide. *Nano Lett.* **17**, 2342-2348 (2017).

27. Joshi, R. K. *et al*. Precise and ultrafast molecular sieving through graphene oxide membranes. *Science* **343**, 752-754 (2014).

28. Qian, Y. *et al*. Enhanced ion sieving of graphene oxide membranes via surface amine functionalization. *J. Am. Chem. Soc.* **143**, 5080-5090 (2021).

29. Ran, J. *et al*. Non-covalent cross-linking to boost the stability and permeability of graphene-oxide-based membranes. *J. Mater. Chem. A* **7**, 8085-8091 (2019).

30. Hirunpinyopas, W., Iamprasertkun, P., Bissett, M. A. & Dryfe, R. A. W. Tunable charge/size selective ion sieving with ultrahigh water permeance through laminar graphene membranes. *Carbon* **156**, 119-129 (2020).

31. Lu, Z. *et al*. Self-crosslinked MXene ($Ti_3C_2T_x$) membranes with good antiswelling property for monovalent metal ion exclusion. *ACS Nano* **13**, 10535-10544 (2019).

32. Ding, L. *et al*. Effective ion sieving with $Ti_3C_2T_x$ MXene membranes for production of drinking water from seawater. *Nat. Sustain.* **3**, 296-302 (2020).
